# Escape from X-inactivation in twins exhibits intra- and inter-individual variability across tissues and is heritable

**DOI:** 10.1371/journal.pgen.1010556

**Published:** 2023-02-21

**Authors:** Antonino Zito, Amy L. Roberts, Alessia Visconti, Niccolo’ Rossi, Rosa Andres-Ejarque, Stefano Nardone, Julia S. El-Sayed Moustafa, Mario Falchi, Kerrin S. Small

**Affiliations:** 1 Department of Twin Research & Genetic Epidemiology, King’s College London, London, United Kingdom; 2 St John’s Institute of Dermatology, Faculty of Life Science & Medicine, King’s College London, London, United Kingdom; 3 Division of Endocrinology, Diabetes, and Metabolism, Department of Medicine, Beth Israel Deaconess Medical Center, Harvard Medical School, Boston, Massachusetts, United States of America; University of Pennsylvania, UNITED STATES

## Abstract

X-chromosome inactivation (XCI) silences one X in female cells to balance sex-differences in X-dosage. A subset of X-linked genes escape XCI, but the extent to which this phenomenon occurs and how it varies across tissues and in a population is as yet unclear. To characterize incidence and variability of escape across individuals and tissues, we conducted a transcriptomic study of escape in adipose, skin, lymphoblastoid cell lines and immune cells in 248 healthy individuals exhibiting skewed XCI. We quantify XCI escape from a linear model of genes’ allelic fold-change and *XIST*-based degree of XCI skewing. We identify 62 genes, including 19 lncRNAs, with previously unknown patterns of escape. We find a range of tissue-specificity, with 11% of genes escaping XCI constitutively across tissues and 23% demonstrating tissue-restricted escape, including cell type-specific escape across immune cells of the same individual. We also detect substantial inter-individual variability in escape. Monozygotic twins share more similar escape than dizygotic twins, indicating that genetic factors may underlie inter-individual differences in escape. However, discordant escape also occurs within monozygotic co-twins, suggesting environmental factors also influence escape. Altogether, these data indicate that XCI escape is an under-appreciated source of transcriptional differences, and an intricate phenotype impacting variable trait expressivity in females.

## Author summary

The difference in the number of X-chromosomes between mammalian males and females is compensated by a process known as X-chromosome inactivation (XCI), which turns off one of a female’s X chromosomes. XCI is incomplete: some sections of the silenced X chromosome escape inactivation. The ’escape’ is complex, and can vary across tissues and potentially across individuals. Because the X chromosome is enriched of genes with immune and neurological functions, this phenomenon has high biomedical relevance. We studied the extent to which escape occurs and varies across tissues and individuals in a large population of twins. We identify novel candidate escape genes, and genes whose escape is specific to a tissue or immune cell type. There is also substantial variability in escape across individuals. Using data from twins, which enable the assessment of the influence of genetics and environment on a trait, we found that both genetic and environmental factors influence escape. Our results allow detailed characterization of escape, and suggest that escape may influence disease risk and phenotype differences between the sexes, and within females.

## Introduction

The X chromosome is a paradigmatic genetic model [1]. It carries >1000 genes, representing >5% of the haploid human genome. It is differentially inherited between the sexes. The unequal X-linked transcriptional dosage between the sexes is partially compensated by random silencing of one X in each female somatic cell [2]. This process, known as X-chromosome inactivation (XCI), involves synergistic DNA-RNA-protein interactions that mediate heterochromatinization of the X designated for inactivation [1,3,4] (known as "Barr Body" [5]). Non-coding RNAs play key roles in XCI. The master long non-coding (lnc) RNA *XIST* spreads in *cis* along the inactive X chromosome (Xi) and promotes a progressive epigenetic silencing [4,6,7]. However, XCI is incomplete, with over 15% of X-genes reported to escape silencing and exhibit expression from both parental alleles within a diploid cell [8,9]. Mary Lyon predicted that genes with Y-homologues (e.g. pseudo autosomal regions (PARs)), are naturally dosage compensated and thus expected to escape [10]. Today, most known escapees lack functional Y-homologues, thus being a potential source of sexual dimorphism [9,11]. Chromosome X is enriched for genes with immune- and neuro-modulatory functions [12,13]; changes in escape may thus underpin not only sexual dimorphism, but also phenotypic and disease risk variability across females [12,14–16]. Despite its biomedical relevance, the inter-individual variability of escape at population level, and across cells and tissues within an individual, has not been systematically examined. Furthermore, the extent to which genetics and environment influence the escape remains largely undefined.

Our current knowledge of XCI escape in humans largely rely on conventional studies of male/female expression ratio, human/mouse hybrid cells, and epigenetic marks [8,11,17,18]. In most females, the random nature of XCI results in expression of both X-linked alleles at a tissue level. This limits the ability to distinguish mono- from biallelic expression and thus identification of escape [9]. To circumvent the problem, strategies like single-cell analyses (e.g. scRNAseq) or sex comparison have been used to infer escape [9,11,19,20]. However, scRNAseq is infeasible for large cohorts and is limited to highly and consistently expressed genes due to allelic drop-out and transcriptional burst, which both can inflate monoallelic expression ratio. On the other hand, sex differences may not directly reflect the allelic expression ratio of X-genes in a tissue. These limitations can be circumvented by using tissue samples exhibiting skewed XCI patterns–a common event in the female population [21–25]–which, as opposed to random XCI, enable detection and measurement of escape directly in skewed females (Note A in S1 Appendix). This strategy has been employed, but either sample sizes were limited (e.g. single GTEx donor), or the study relied on arrays and other indirect biological models [8,11,26].

Here, we characterize XCI escape using paired bulk RNAseq and DNAseq data in a multi-tissue dataset sampled from 248 skewed female twins of the TwinsUK bioresource [27]. We investigate escape prevalence and variability across adipose and skin tissue, lymphoblastoid cell lines and purified immune cells (monocytes, B-cells, T-CD4^+^, T-CD8^+^ and NK cells), and individuals. We identify novel genes exhibiting tissue- and immune cell type-specific escape, and genes escaping XCI with high variability across tissues and individuals. We observe that escape varies across tissues and immune cells within an individual and across individuals, a phenomenon with high biomedical relevance. Using twins, we demonstrate that regulation of XCI escape has both heritable and environmental components, implying a complex interplay between genetic and non-genetic factors.

## Results

### Escape from X-inactivation is a prevalent phenotype in both solid and blood-derived tissues

**Fig 1:**
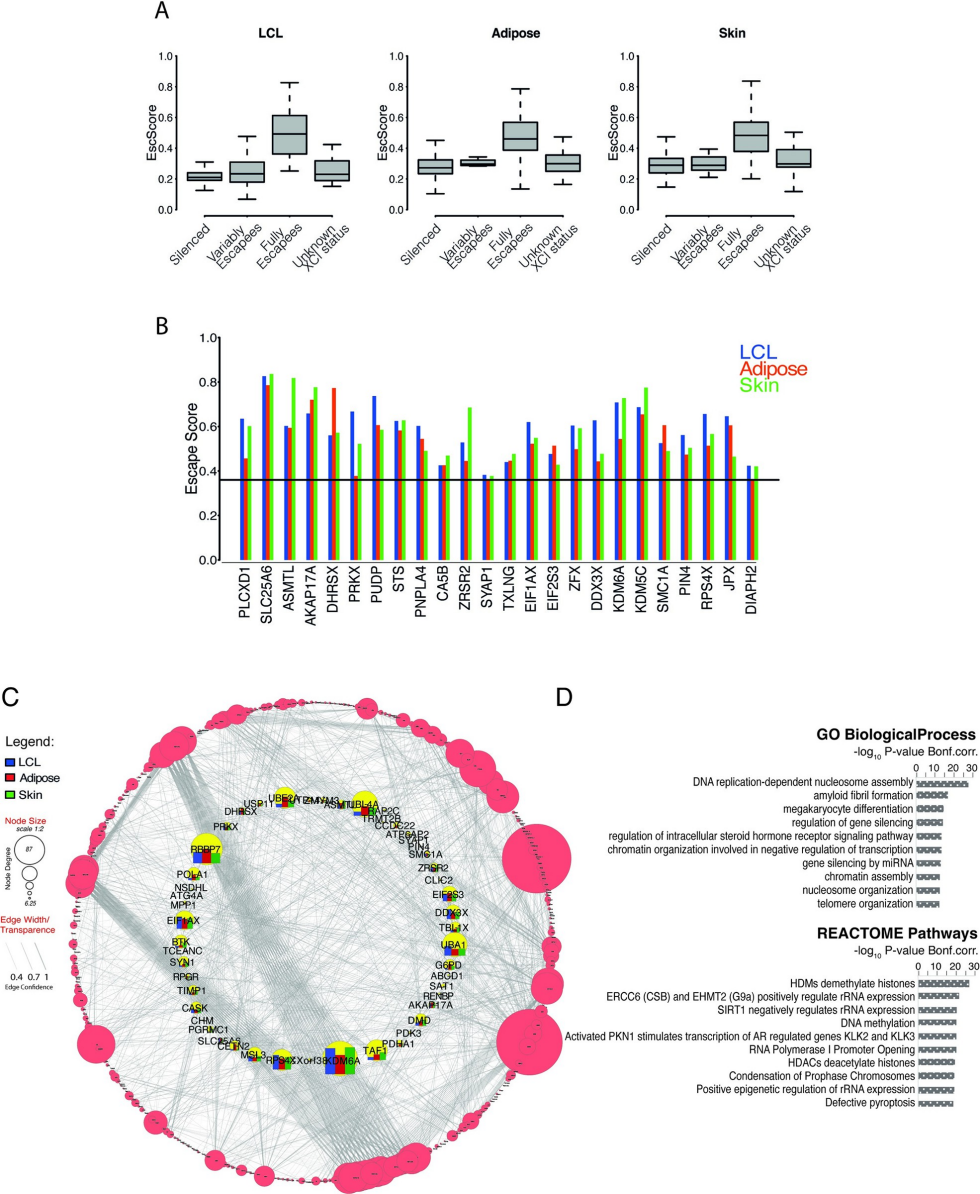
(A) EscScore distinguishes between silenced and escape genes. Boxplots show the distribution of median EscScore values (across skewed samples) of genes with different previously annotated XCI status [30]. (B) Genes exhibiting constitutive XCI escape in all three tissues. Barplots show the tissue-specific gene’s EscScore. Horizontal line denotes the EscScore cutoff to call XCI escape. (C-D) PPI, Gene Ontology (GO) and Pathway analysis. (C) PPI network between protein-coding X-genes escaping XCI in at least one tissue and a reference map of human proteome interactome [32]. The network inner circle shows protein-coding escape genes characterized within the proteome interactome. The bars for the genes in the inner circle show the tissue-specific gene’s EscScore. The network outer circle shows proteins from the reference proteome [32] that have (i) at least 1 interaction with our escapee genes and (ii) edge confidence score ≥0.4. The size of each node reflects the number of interactions (edges). (D) GO and REACTOME Pathways analysis of PPI network. The top 10 Biological Processes and REACTOME terms are shown.

We quantified escape in multiple tissues concurrently sampled from female twins of the TwinsUK cohort [27]. We determined XCI patterns using the gene-level *XIST* allele-specific expression (*XIST*_ASE_) from paired RNAseq and DNAseq data [7,28,29]. From over 2200 tissue samples interrogated, we obtained *XIST*_ASE_ calls for 522 LCLs, 101 whole-blood, 421 adipose, and 373 skin samples. In samples exhibiting skewed XCI (*XIST*_ASE_≤0.2 or ≥0.8, Methods) including 166 LCLs (32%), 26 whole-blood (26%), 57 adipose (14%), and 64 skin (17%) samples, the levels of escape of each X-linked gene were measured using a metric—herein referred to as Escape Score or ’EscScore’—derived from the gene’s allelic fold-change adjusted for the sample’s XCI skew (Methods). EscScore values range from 0 (no escape, monoallelic expression) to 1 (full escape, equal expression from inactive (Xi) and active X (Xa)). We interrogated a total of 551 genes, of which 85% are protein-coding and 15% non-coding RNA genes. Based on a publicly available catalogue of XCI statuses [30] (’Balaton’s list’), our interrogated genes were categorized as XCI-silenced (n = 326), fully or mostly escaping XCI (n = 52), or variable escapees (n = 23). Variable escape refers to genes whose escape is variable across cells, tissues, or individuals [8,11,30]. We also included a subset of 41 genes whose XCI status was reported as discordant across studies or undefined [30]. The summary statistics indicated that EscScore differs between different categories (Table A in S1 Appendix). In all tissues, the EscScore of genes annotated as fully or mostly escaping XCI significantly differed from genes annotated as either silenced or variable escapees (Fig 1A and Table B in S1 Appendix), supporting the reliability of our escape metric to discriminate different XCI statuses.

Next, we conducted additional testing of EscScore, identified genes escaping XCI in our dataset, and benchmarked our call set with other studies. For X-genes annotated as silenced [30], the average EscScore was 0.26, 0.33, and 0.34 in LCLs, adipose and skin tissues, respectively, whose mean is 0.31 and median 0.33. When including all X-linked genes interrogated, we detected average EscScore of 0.32, 0.36, and 0.37 in LCLs, adipose and skin, respectively, whose mean is 0.35 and median 0.36. We tested several EscScore cutoffs by comparing resulting gene classification with the Balaton’s list of XCI status [30]. We found that a threshold of 0.36 resulted to both overall higher reproducibility of gene calls and lower discordance with previously annotated XCI status (Table C in S1 Appendix). Based on these observations, we classified genes with a median (across ≥3 skewed tissue samples) EscScore≥0.36 as escapees in that tissue, while genes with EscScore<0.36 as silenced. In line with current knowledge, most X-genes are subject to XCI in all tissues (84% in LCLs, 74% in adipose, 71% in skin). We observed a higher incidence of escape in solid tissues than LCLs, with 16%, 26% and 29% of genes escaping XCI in LCLs, adipose and skin tissues, respectively. Altogether, 157 X-genes exhibited escape in at least one tissue in our dataset (S1 Table). Expectedly, PAR-linked genes escaped XCI. We used a hypergeometric test to assess overlap between the Balaton’s list [30] and our list of escapees, and found significant overlap (N = 50; *P*≤0.05). Among our escape calls, about 60 genes retain a Y-pseudogene or Y-homologue, and 13 are PAR-linked, supporting their escape [30]. For a more comprehensive comparison, we merged the Balaton’s list of escapees with additional external lists of escapees including (i) the GTEx XCI survey [11]; (ii) Katsir et al. [20]; (iii) Shvetsova et al [21]; (iv) Garieri et al. [19]; (v) Sauteraud et al. (predicted from GEUVADIS) [16]. We found that 60% (N = 95) of our escapees overlapped with this unified list of escapees, while the remaining (N = 62) represent novel calls in our study. Notably, 31% of our novel escape calls are annotated as ncRNA, while the remaining are protein-coding. We establish the first evidence that the lncRNA *AL683807*.*1* (ENSG00000223511) escapes XCI. *AL683807*.*1* is PAR1-linked, explaining its escape ability. We examined the chromosomal distribution of escape genes and confirmed a higher escape incidence on the short arm [31] (Note B in S1 Appendix and Fig C in S1 Appendix). Altogether, these data further support the suitability of our study design. We show that in our data, escape is more prevalent in adipose and skin than LCLs, and is wider than previously reported estimates.

### Escape from X-inactivation exhibits both constitutive and tissue-specific patterns

Presently, the extent to which tissue-specific escape occurs in humans is unclear. We used the gene’s median EscScore (across ≥3 tissue samples) as a measure for tissue-specific levels of escape. We found significant differences across tissues (Kruskal-Wallis (’KW’) *P-value<*10^−10^; Fig D in S1 Appendix), suggesting tissue differences in escape. Using a subset of 213 genes with EscScore available in all tissues (S2 Table), we identified 24 genes exhibiting escape in both LCLs and solid tissues (Fig 1B), suggesting constitutive escape. We observed that tissue-specific EscScore remained below 80% in most cases, in line with data showing that Xi/Xa expression ratio would not exceed 80% [11]. Notably, *PLCXD1*, *ASMTL*, *DHRSX*, *SLC25A6* and *AKAP17A* are PAR-linked. We show that *PUDP* and *PIN4*, whose escape status have remained so far unclear, show constitutive escape in all tissues. Among the constitutive escapees, there are the highly biomedically-relevant genes *DDX3X*, *KDM5C* and *KDM6A*, whose escape may contribute to lower cancer incidence in females than males [14]. We also observed that *PRKX*, *PUPD*, *DDX3X* and *JPX* each had significantly different escape between tissues (KW *P*≤0.05). We confirmed the escape status of *CLIC2* in skin, as identified in GTEx [11], and also found it escapes in adipose in our data. Tissue-specificity of escape was further supported by identification of 49 genes exhibiting escape restricted to a single tissue. Notably, 24 of these, of which 2 non-coding RNAs and 22 protein-coding genes, are novel escape calls (S3 Table).

We investigated whether the escapees may interact with other factors and be involved in biological processes. To address this, we selected genes exhibiting escape in at least one tissue and conducted protein-protein interaction network analysis using a recent human protein interactome as reference [32]. We found that protein-coding escape genes interact with other factors on a genome-wide scale (Fig 1C). Gene ontology analysis revealed that members of this proteome network are involved in distinct biological processes such as epigenetic regulation by chromatin assembly and nucleosome organization, and regulation of steroid hormone signaling. These data were supported by REACTOME pathway analysis which revealed multiple pathways for epigenetic control of genes, such as histone methylation and acetylation, and DNA methylation (Fig 1D and S4 Table). Altogether, these data suggest that escape is shaped by an interplay of tissue-shared and tissue-specific factors, and participates in genome-wide interactions involved in varied biological processes.

### Escape from X-inactivation exhibits intra- and inter-individual variability

**Fig 2:**
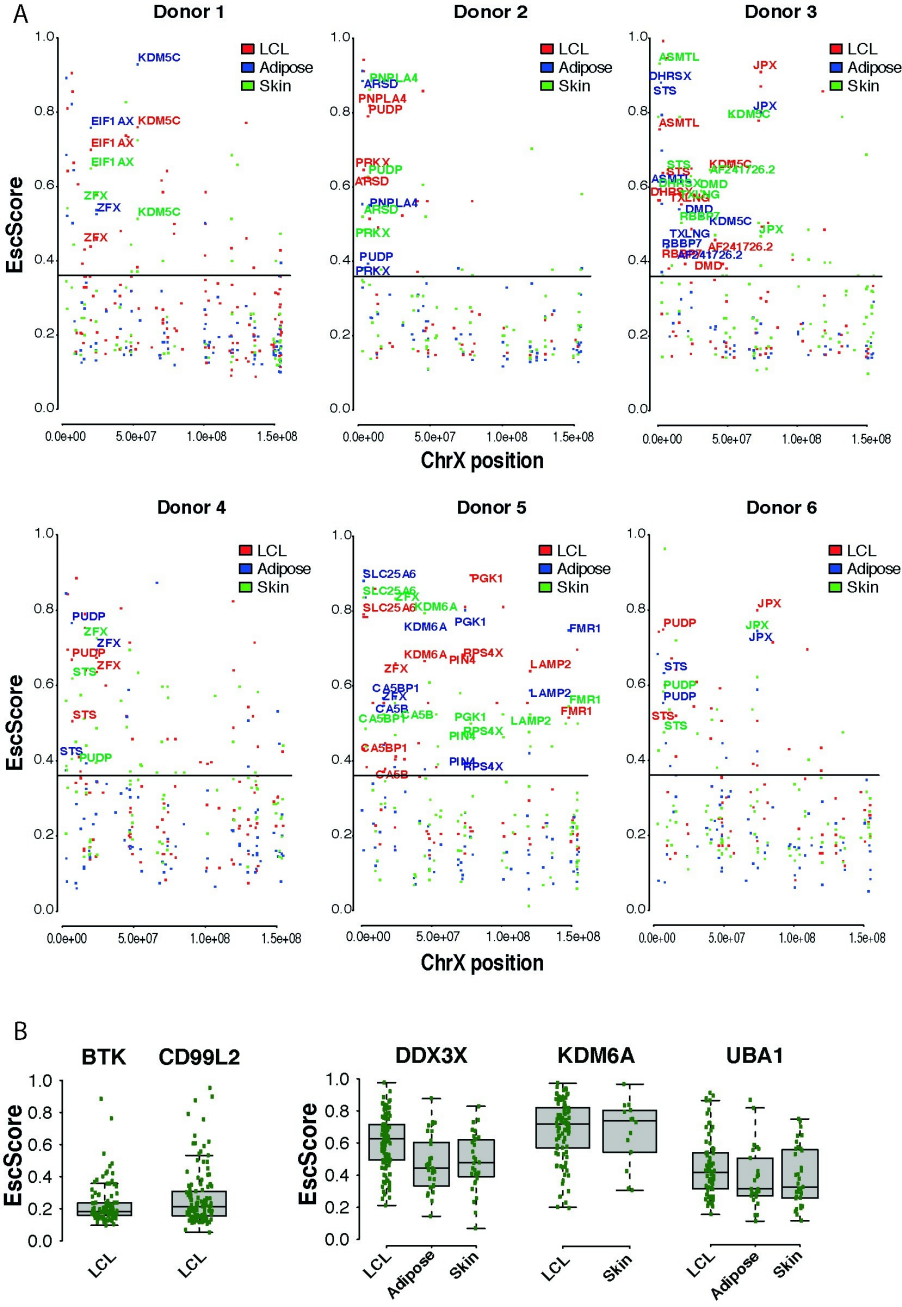
(A) Analysis of intra-individual EscScore variation within each of the 6 female donors exhibiting skewed XCI in all tissues. Each dot shows the gene’s EscScore in each tissue. Genes with EscScore≥0.36 in all three tissues within a donor are colored in red (LCLs), blue (adipose), green (skin). (B) Representative subset of genes to assess inter-individual variation in EscScore. Each green dot shows the gene’s EscScore in a tissue sample. *BTK* and *CD99L2* show consistent EscScore in LCLs across individuals. *DDX3X*, *KDM6A* and *UBA1* show inter-individual variability of EscScore in LCLs and a solid tissue. Similar plots for other genes are shown in S1 and S2 Figs.

Understanding the extent to which escape varies across tissues within an individual is of high biological interest. We investigated this phenomenon in 6 donors, each exhibiting skewed XCI in LCLs, adipose and skin. This strategy was employed in the GTEx survey using a single skewed female donor [11]. Within each donor, we examined all genes with available EscScore. We identified genes exhibiting escape (EscScore≥0.36 in a tissue within a donor) in 1, 2 or 3 tissues and found that their prevalence varied between donors (Fig 2A and Table D in S1 Appendix). Occurrence of genes escaping XCI in all tissues in multiple donors suggests shared regulatory mechanisms across tissues and individuals. Genes exhibiting such a behaviour included the zinc-finger protein *ZFX* and the histone demethylase *KDM5C* which is linked to intellectual disability and autism [33,34]. We also observed genes escaping XCI in all tissues but in only 1 of the 6 donors. Examples are the leukaemia-protecting histone demethylase *KDM6A* [14,35], *FMR1*, a gene linked to Fragile-X and learning disability [36], and the Duchenne muscular dystrophy gene *DMD* [37]. In parallel, we found instances of genes whose EscScore widely ranged across tissues within a donor, as *ASMTL* which escaped XCI in all tissues with EscScore ranging from 0.6 to over 0.8. *ASMTL*’s behavior was also highlighted in GTEx [11]. Aside from these cases, most of the interrogated genes exhibited tissue-restricted escape, supporting the occurrence of tissue-specific factors exerting dominant effects.

To robustly investigate the inter-female diversity in escape, we used a subset of 125 genes escaping XCI in at least 1 tissue, and with EscScore available in at least 10 individuals per tissue. For a given gene, we defined its EscScore to be consistent within a tissue if in at least 80% of individuals the gene’s EscScore lay between ±1 standard deviation from the gene’s average EscScore in the tissue. This strategy revealed both genes with consistent and genes with variable EscScore. We identified 35 genes (~30% of the interrogated genes) showing consistent EscScore across individuals in at least 1 tissue (S1 Fig). Representative examples are *BTK*, a gene involved in the control of lymphocyte maturation, and *CD99L2*, involved in leucocyte homeostasis. Both genes exhibited consistent EscScore across most donors in LCLs (Fig 2B and S5 Table). A subset of 3 genes (*ARHGAP6*, *SAT1*, *RAP2C-AS1*) also exhibited consistency across most donors in both LCLs and a solid tissue (S1 Fig). Genes exhibiting inter-female variability in EscScore in multiple tissues (S2 Fig) accounted for about 50% of the interrogated genes. Examples are *DDX3X*, *KDM6A* and *UBA1*, which exhibited variability in LCLs and at least one solid tissue (Fig 2B and S5 Table). Interestingly, inter-female variability occurred more frequently in solid tissues (62% of cases) than LCLs (38% of cases). Altogether, these data are indicative of complex escape patterns. Variable escape across females complements with and may be driven by variable escape across tissues and cells within a female. Inter-female variation has high biomedical relevance as it may underlie predisposition to and manifestation of X-linked traits.

### Escape from X-inactivation exhibits immune cell type-specificity

**Fig 3:**
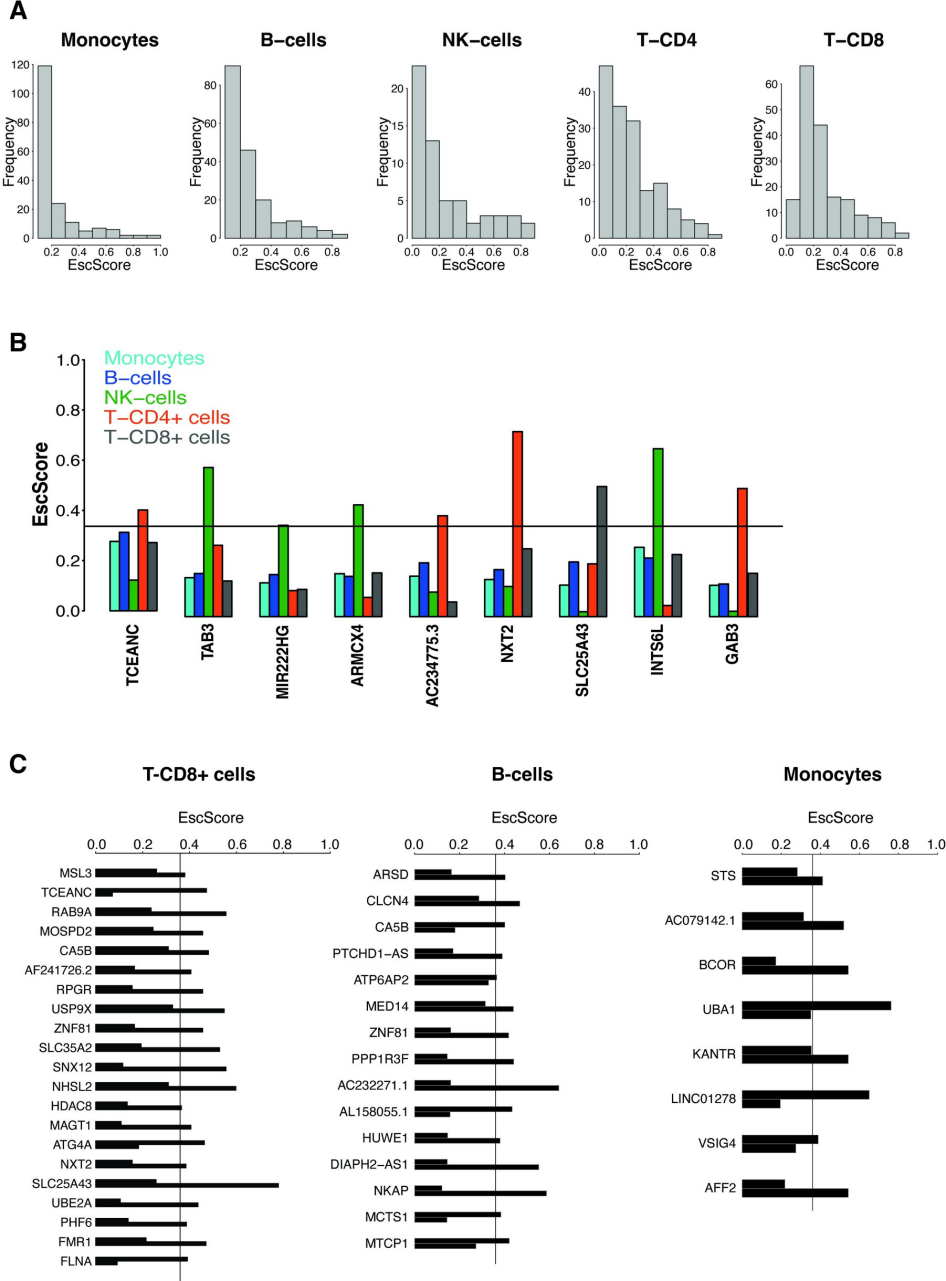
(A) Distribution of EscScore(s) in each immune cell type. (B) Genes exhibiting XCI escape restricted to a single immune cell type. Barplots show the immune cell type-specific gene’s EscScore. (C) Genes exhibiting discordant XCI status (escaping XCI only in one co-twin) between the two MZ co-twins. The two bars shows the gene’s EscScore in an immune cell type (T-CD8^+^ cells, B-cells and monocytes) in the two co-twins.

Females have a higher risk of autoimmune disease than males, and such risk may correlate with increased X-dosage [38,39]. This has raised the hypothesis that XCI escape may contribute to autoimmunity [12,40]. The extent to which escape varies across different immune cells within an individual is not well known. We addressed this question by interrogating 257 X-genes in multiple immune cell types purified from two identical co-twins (Fig E in S1 Appendix). Monocytes, B-cells and T-CD8^+^ cells were available from both co-twins, while T-CD4^+^ and NK-cells from one co-twin. Per each immune cell type, when data were available from both co-twins, we calculated the average gene’s EscScore across the 2 co-twins as a proxy for immune cell type-specific escape. We observed differences between cell types in the average EscScore (Av.EscScore_Monocytes_ = 0.24; Av.EscScore_B-cells_ = 0.27; Av.EscScore_T-CD4+_ = 0.24; Av.EscScore_T-CD8+_ = 0.28; Av.EscScore_NK-cells_ = 0.25; KW *P*≤0.01; Fig 3A). These results were consistent when comparison was limited to a subset of 53 genes with EscScore data available in all cell types (S6 Table; Av.EscScore_Monocytes_ = 0.245; Av.EscScore_B-cells_ = 0.27; Av.EscScore_T-CD4+_ = 0.26; Av.EscScore_T-CD8+_ = 0.285; Av.EscScore_NK-cells_ = 0.245; *P*≤0.01). The incidence of escape varied between cell types, being 15% in monocytes, 20% in B-cells, 22% in T-CD4^+^, 25% in T-CD8^+^, and 29% in NK-cells. Thus, in line with current knowledge, most X-genes are subject to XCI in immune cells. In parallel, our data indicate that escape is heterogeneous across immune cell types, with overall higher incidence in lymphocytes than monocytes. To investigate intra-lineage variation, we compared the EscScore(s) between lymphoid cell types and also found substantial differences (*P*≤0.01), indicating intra-lineage variation. Among the 53 genes with EscScore data available in all cell types (S6 Table), we identified 12 genes (*ARSD*, *PRKX*, *PUDP*, *CA5B*, *AP1S2*, *ZFX*, *USP9X*, *DDX3X*, *CASK*, *KDM6A*, *JPX*, *DIAPH2*) escaping XCI in at least three immune cell types. *CASK* is a novel candidate escapee. The genes *PRKX*, *ZFX*, *JPX* and *DIAPH2* escaped XCI in all 5 immune cell types, in line with their behavior as constitutive escapees across tissues. For most of these genes, the escape status in immune cells is a novel finding. Interestingly, *KDM6A* exhibited highest EscScore in T-CD8^+^ cells, possibly because of its roles in T-cells control [35]. We identified 9 genes exhibiting escape restricted to one immune cell type, supporting immune cell type-specific factors (Fig 3B). Intriguingly, immune cell type-specific events were restricted to lymphocytes but not monocytes. This might suggest differences between lymphoid and myeloid lineages, and aligns with evidence of increased X-linked biallelic expression in lymphocytes [41]. We also assessed the skewed LCL samples which were also available from both co-twins. We found that in these two donors, about 21% and 22% of genes with available data had EscScore≥0.36. These values are similar to the incidence of escape we detected in lymphoid cells (B, T-CD4^+^ and T-CD8 cells). Altogether, these data indicate that escape varies between immune cell types within an individual. Presumably, this heterogeneity is driven by mechanisms with immune cell type-specific effects.

### Escape from X-inactivation is influenced by heritable and environmental factors

**Fig 4:**
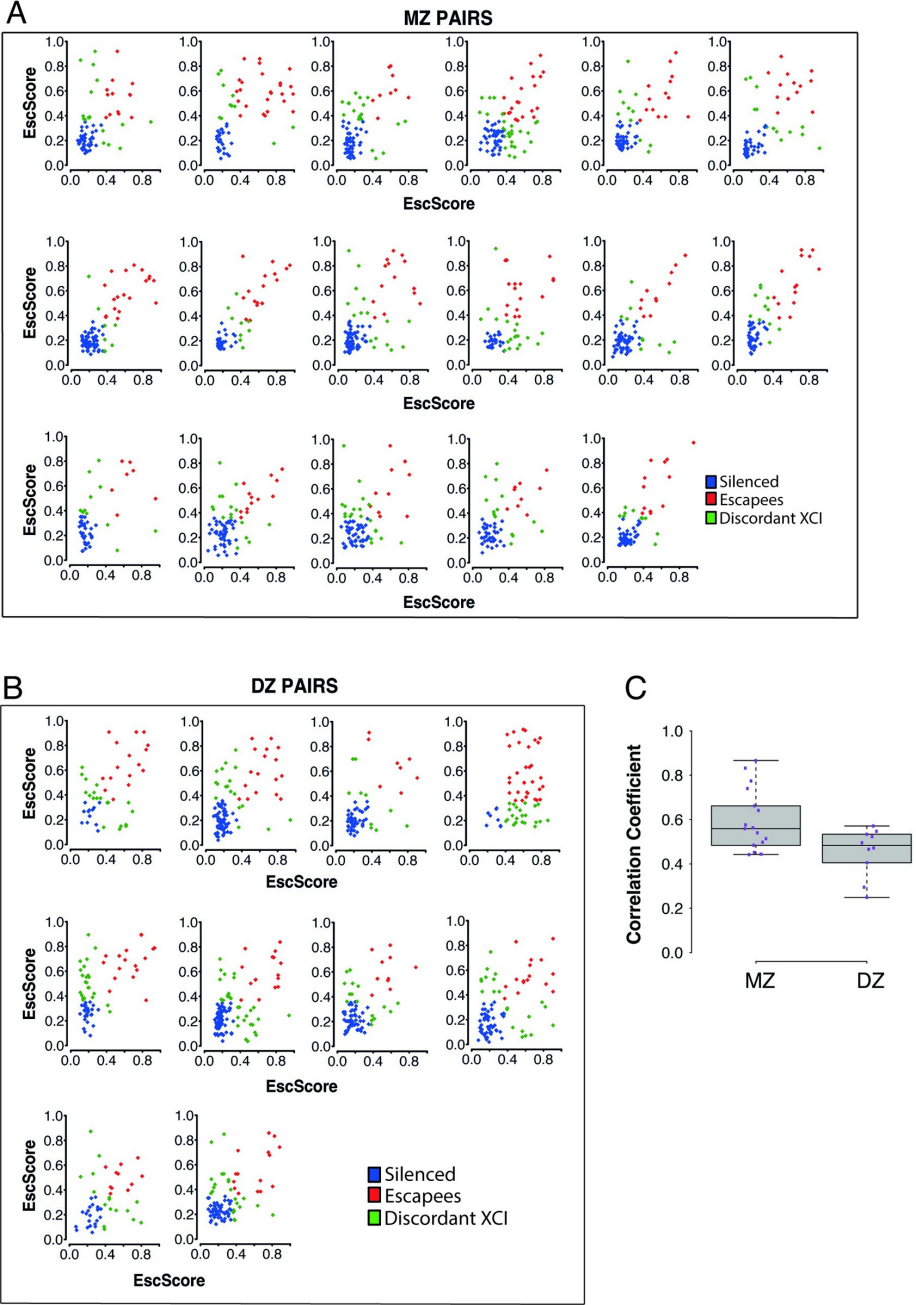
Scatterplots of EscScore(s) of genes (≥5) with data available in both co-twins of a pair. A total of 27 complete twin pairs (both co-twins exhibiting skewed XCI in LCLs) were used. Each dot represents a gene, colored in blue if silenced in both co-twins, red if escaping XCI in both co-twins, and green if exhibiting discordant XCI (escaping only in one co-twin). (A) Monozygotic (MZ) twin pairs (N = 17); (B) Dizygotic (DZ) twin pairs (N = 10). (C) Boxplots of coefficients of correlation between EscScore(s) in the two co-twins of each pair.

Twin studies are a unique strategy to assess the contribution of genetic factors to complex traits. Using 27 complete twin pairs (17 monozygotic (MZ or identical); 10 dizygotic (DZ or fraternal)), we quantified the concordance in the escape in LCLs between co-twins and compared such concordance between MZ and DZ twins. We correlated the EscScore (using ≥5 genes) between co-twins of each pair (Fig 4A and 4B), and found that the average correlation across MZ and DZ twins was 0.6 and 0.46, respectively (*ρ*′*s* t-test, *P*≤0.05; Fig 4C). These data indicate that MZ share significantly more similar escape than DZ twins. To support this finding, we examined each interrogated gene in a twin pair, and observed higher rates of discordant XCI (gene escaping XCI only in one of the two co-twins) between DZ than MZ twin pairs (Av.Disc.Rate_DZ_ = 27.1%; Av.Disc.Rate_MZ_ = 19.5%). These data suggest a significant genetic component of escape, in line with a previous report on MZ twins [17], and the higher similarity in skewing between MZ than DZ twins in blood-derived tissues [23,28]. In parallel, discordance between MZ twins suggests environmental influences. To gain insights at the cell type level, we next examined the concordance of EscScore in immune cells between two MZ co-twins, and observed significant correlations (*ρ*_monocytes_ = 0.8; *ρ*_B-cells_ = 0.68; *ρ*_T-CD8+_ = 0.6; *P*<1e-10). Genes with discordant XCI status were observed in all three immune cell types, and their prevalence differed between cells ranging from 6.4% in monocytes to 11% in B-cells, and 18% in T-CD8^+^ cells. Interestingly, the genes *CA5B* and *ZNF81* exhibited discordant XCI between the two co-twins in both T-CD8^+^ and B-cells. In all other cases, discordant XCI events concerned distinct gene subsets in distinct immune cell types (Fig 3C and S7 Table). Taken together, our data indicate that genetic and environmental factors may interplay to regulate XCI escape. Variability between immune cell types may also suggest an immune cell type-specific response to environmental influences.

## Discussion

In this study, we investigated escape in tissues and immune cells using paired transcriptomic and genotype data from nearly 250 female twins from the TwinsUK bioresource [27]. The large sample size and strategy of using bulk tissue samples with skewed XCI as a platform to infer escape [8,9,11,26,42], enabled us to systematically distinguish silenced from escape genes and identify novel candidate escapees, as predicted [9]. While samples with random XCI are a mosaic of cells with either parental X silenced, in skewed samples expression would be mostly restricted to one haplotype. We quantify escape from the residuals of linear model of genes’ aFCs and XIST-based degree of XCI skewing in skewed samples. Our data show the incidence of escape varies across tissues, and is higher in solid than blood tissues. The higher escape rate in solid tissues than LCLs and purified cell types might be a reflection of their higher biological heterogeneity resulting from the more complex cell type composition. Most of our escape calls align with previously annotated XCI statuses, however we identified 62 novel candidate escapees, of which 43 (69%) are protein-coding and 19 (31%) lncRNA genes. Protein network analyses show that X-linked protein-coding genes escaping XCI interact with other proteins on a genome-wide scale, and regulate varied biological processes and pathways such as epigenetic changes and hormone signaling. These data indicate that XCI escape may play genome-wide effects, in line with recent findings on the effects of XCI changes on global proteome [43]. Thus, de-regulated escape caused by mutations or other disrupting events may have complex phenotypical consequences. Future studies are needed to investigate the functional effects of X-autosomal interactome. Our discovery set includes the PAR1 lncRNA *AL683807*.*1*, which escaped XCI in LCLs, in line with GTEx data on the novel transcript ENSG00000223511.6, whose expression is substantially higher in EBV-transformed lymphocytes than all other tissues. The X chromosome is enriched for non-coding RNAs, yet their transcriptional modes and roles are unclear. Due to their unique ability to recruit factors and target a genomic address [1], lncRNAs play critical roles in genome regulation and health. The study of lncRNAs escaping XCI may reveal novel mechanisms of inter-female phenotypic variation and sexual dimorphism.

We identified genes that constitutively escaped XCI in all tissues, and genes with tissue-specific escape. Co-occurrence of both patterns suggests involvement of tissue-shared and tissue-specific determinants. Presumably, genetic variants (e.g. eQTLs) with constitutive or tissue-specific effects, modulate the escape dosages. We found genes with consistent and with heterogeneous EscScore across individuals. We reasoned that genes with key physiological roles may be subject to shared regulation across females. Examples are *BTK* and *CD99L2*, which, in line with their roles in lymphoid cells, exhibited consistent EscScore in LCLs, but inter-female variability in adipose and skin. Thus, the escape behaviour could depend on the gene’s functional context. The tumorigenesis-related genes *DDX3X* and *KDM6A* both escaped XCI, in line with previous studies [11,30,44], and showed inter-female variability in all tissues. These genes may underlie sexual dimorphism in cancer [14], and plausibly their variable escape contributes to inter-female diversity in cancer risk. We found that the histone demethylase *KDM5C* and the transcription factor *ZFX*, escaped in LCLs and solid tissues in the same individual. Other genes manifested more composite behaviour, with escape restricted to either LCLs or solid tissues in an individual, supporting tissue-specificity of escape. Altogether, these patterns highlight the complexity of escape, and anticipate its roles as phenotype modulator. The intra-individual analysis would benefit from a larger sample size, and additional tissue types available from each individual, allowing more comprehensive and precise inferring of the across-tissue escape variability within a subject. Furthermore, the variable nature of RNAseq total read depth introduces differences in allelic read depth across samples, which may contribute to the observed differences, and will impact the total number of genes within a donor with sufficient read depth to analyze escape. Despite the use of established methods, we acknowledge that phasing switch errors can occur over the chromosomal length, a problem that can be solved by the availability of parental genotypes (e.g. [21]).

The X chromosome plays key roles in innate and adaptive immunity [12,31,40,45]. We found that escape differed between immune cell types, with higher incidence in lymphocytes than monocytes. Among 53 genes with data available in all immune cell types, about 8% (*PRKX*, *DIAPH2*, *JPX*, *ZFX*) escaped XCI in all cells, in line with their constitutive escape behavior across tissues. For *ZFX*, this aligns with data showing its involvement in networks for X-linked dosage regulation [46]. 17% of genes (*TCEANC*, *TAB3*, *MIR222HG*, *ARMCX4*, *AC234775*.*3*, *NTX*, *SLC25A43*, *INTS6L*, *GAB3*) exhibited escape restricted to a single immune cell type which was always of lymphoid lineage. We also found significant variation between lymphoid cells. These data indicate immune cell type-specific propensity to escape. Myeloid and lymphoid lineages are subject to distinct regulation during development. Integrated functional and in-silico approaches will be needed to fully address the possibility that cell type-specific factors establish distinct escape dosages across cells within an individual [11]. These data would have multiple biological significance. Firstly, different cell types and possibly single cells, would provide a different contribution to the overall escape dosages in a tissue, establishing an X-linked transcriptional mosaicism throughout the female’s immune system. Secondly, changes in cell type composition or proportion, which may characterize pathological states [47,48], may alter the escape which in turn modifies disease risk. Immune cell type-specific escapees could potentially serve as markers of disease-relevant cells, with applications for diagnostic purposes and design of immunotherapy approaches [49–51]. The extent to which this phenomenon modulates inter-female variability in risk and expressivity of immunological traits will require future larger studies. Despite the availability of multiple immune cell types, these were drawn from only two genetically identical co-twins thus limiting further analysis of between-individual variation in this context.

The extent to which genetics and environment influence escape in humans is unclear. Concordance in methylation-based XCI status between MZ twins supported a dominant model of cis-acting influences [17]. MZ twins share >99% of DNA, age, and multiple environmental traits such as in-utero growth and early life. Variable escape may affect MZ twins differently, leading to different trait expressivity. We found significantly more similar EscScore between MZ than DZ co-twins. Congruently, we found overall higher rates of discordant XCI between DZ than MZ co-twins. These data demonstrate a solid contribution owing to genotype, but also that DNA does not fully explain such concordance patterns. Thus, escape has both heritable and environmental components, in line with current knowledge on complex traits. An interplay between QTLs [52,53], differential epigenetic control of parental alleles [1], and gene-environment effects may ultimately modulate the allelic expression and propensity to escape. These effects might have cell type- and tissue-specific components, and underlie intra- and inter-female variation. The inter-female variability align with population differences in dose compensation [26,54], supporting involvement of genetic factors. Cis-acting variation may also model Xi vs Xa haplotype expression across tissues, leading to intra-individual variation [11]. Identification and functional characterization of such genetic and environmental factors can aid understanding what drives inter-female variation in trait expressivity, disease risk, and sex differences.

The present study contributes a detailed characterization of escape in humans using a large multi-tissue transcriptomic twin dataset and demonstrates extensive variability in escape between individuals and tissues. Given the paradigmatic roles of the X chromosome in epigenetics and clinical genetics, a full understanding of XCI escape has implications on epigenetic research and therapeutics. Therapeutics may include genetic counselling and design of treatments for X-linked conditions. Despite nearly 60 years after Mary Lyon’s landmark intuition on escape, a lot is yet to be learned. Future large-scale studies that combine biomedical records and functional assays will be critical to disentangle the breadth of variability of escape from XCI in humans and characterize its phenotypic impact.

## Materials and methods

### Ethics statement

This project was approved by the research ethics committee at St Thomas’ Hospital (London, UK). Volunteers received detailed information sheet regarding all aspects of the research, gave informed consent and signed an approved consent form prior to biopsy and to participate in the study. See Materials and Methods (section on Sample collection) for further details.

### Sample collection

The study included 856 female twins from the TwinsUK registry [27,55] who participated in the MuTHER study [52]. Study participants included both monozygotic (MZ) and dizygotic (DZ) twins, aged 38–85 years old (median age = 60). All subjects are of European ancestry. Peripheral blood samples were collected and lymphoblastoid cell lines (LCLs) generated via Epstein-Barr virus (EBV) mediated transformation of B-lymphocytes. Punch biopsies of subcutaneous adipose tissue were taken from a photo-protected area adjacent and inferior to the umbilicus. Skin samples were obtained by dissection from punch biopsies. Adipose and skin samples were weighed and frozen in liquid nitrogen.

### DNA sequencing data and variant calling

Details on 30X whole genome sequencing (WGS) sample and library preparation, clustering and sequencing have been reported elsewhere [56]. The DNA sequencing reads were stored offsite pre-mapped to the X chromosome with Illumina’s ISIS Analysis Software v.2.5.26.13 [57]. For the purpose of this project, all individuals were female, reads premapped to chrX,Y and unmapped reads were extracted from the original ISIS alignment and realigned to the GRCh38 X chromosome reference sequence using BWA-MEM in SpeedSeq v0.1.2 [58]. Base quality score recalibration (BQSR) was performed in GATK v4.1.6 [59]. Following this, DNA variant calling was performed using the gold-standard workflow in GATK v4.1.6 [59]. This included implementation of HaplotypeCaller to call germline variants, GenomicsDBImport to create a unified gVCF repository, and GenotypeGVCFs for joint genotyping to produce a multi-sample variant call set. Variants with a VQSLOD (variant quality score odd-ratio) corresponding to a truth sensitivity of <99.9% and with a HWE (Hardy-Weinberg equilibrium) *P-value* <1e-6 were removed. Data quality checks were further performed with VCFtools[60] to check levels of transition/transversion ratio. Dataset comprised 621 female samples. For individuals with available RNA-seq but unavailable genotypes, chrX DNAseq data were retrieved from the UK10K project [61].

### RNA sequencing data

The Illumina TruSeq sample preparation protocol was used to generate cDNA libraries for sequencing. Libraries were sequenced on a Illumina HiSeq2000 machine and 49 bp paired-end reads were generated [55]. Samples that failed library preparation (according to manufacturer’s guidelines) or had less than 10 million reads were discarded. As all individuals were female, for this manuscript RNA-seq reads were aligned to a Y-masked [62] GRCh38 reference genome using STAR v.2.7.3 [63]. Properly paired and uniquely mapped reads with a MAPQ of 255 were retained for further analyses.

### Purified immune cell RNA-sequencing data

Monocytes, B, T-CD4^+^, T-CD8^+^ and NK cells were purified using fluorescence activated cell sorting (FACS) from two monozygotic twins exhibiting skewed XCI patterns in LCLs. Gating strategy for cell sorting is described in Fig E in S1 Appendix. Total RNA was isolated and cDNA libraries for sequencing were generated using the Sureselect sample preparation protocol. Samples were then sequenced with the Illumina HiSeq machine and 126 bp paired-end reads were generated. Adapters and polyA nucleotide sequences were trimmed using trim_galore v.0.6.3 and PrinSeq tools v.0.20 [64]. RNA-seq reads were aligned to Y-masked [62] GRCh38 reference genome using STAR v.2.7.3 [63]. Properly paired and uniquely mapped reads were retained for further analyses.

### Correction of RNA-sequencing mapping biases

To eliminate mapping biases in RNA-seq, the WASP pipeline for mappability filtering [65] in STARv2.7.3 [63] was used. In each read overlapping a heterozygous SNP, the allele is flipped to the SNP’s other allele and the read is remapped. Reads that did not remap to the same genomic location are flagged as owing to mapping bias and were discarded.

### Haplotype phasing and measurement of gene-level haplotype expression

WGS genomes were read-back phased using recent SHAPEIT2 implementation [66] that takes advantage of the phase information present in DNA-seq reads. Subsequently, phASER v.0.9.9.4 [67,68] was used for RNAseq-based read-backed phasing and to generate gene-level haplotype expression data. Only reads uniquely mapped and with a base quality ≥10 were used for phasing. Using haplotype expression data, the gene’s ASE in each sample can be calculated as follow:

$$ASE_{g,s}=\frac{AC_{g,s}}{TC_{g,s}}Gene's\;Allele‐Specific\;Expression\;(ASE)$$ [1]

Where, for a biallelic gene:

A = haplotype A;

B = haplotype B;

AC_g,s_ = RNAseq allelic count at haplotype A of gene g in sample s;

BC_g,s_ = RNAseq allelic count at haplotype B of gene g in sample s;

TC_g,s_ = Total RNAseq allelic read depth at gene g in sample s;

TC_g,s_ = AC_g,s_ + BC_g,s_.

The gene ASE values range from 0 to 1, with 0 and 1 indicating monoallelic expression and 0.5 indicating completely balanced haplotypic expression. From [1], it follows:

$$\frac{AC_{g,s}}{TC_{g,s}}+\frac{BC_{g,s}}{TC_{g,s}}=1$$ [2]

To quantify gene silencing and gene escape, the effect size of allelic imbalance in expression for each gene in each sample was calculated as allelic fold change (aFC), that is the ratio between the allele with lower RNAseq count and the allele with the higher RNAseq count, as similarly used in a previous study [69]:

$$aFC_{g,s}=\frac{Min(AC_{g,s},BC_{g,s})}{Max(AC_{g,s},BC_{g,s})}aFC\;of\;gene\;g\;in\;sample\;s$$ [3]

aFC values range from 0 to 1, with 0 indicating monoallelic expression (full gene silencing) and 1 completely balanced haplotypic expression (full escape). For the purpose of our study, a gene’s aFC can be interpreted as Xi/Xa expression ratio. The Xi is assumed to be the allele with the lower RNA-seq count, while the Xa the allele with higher RNA-seq count.

### Quantification of XCI skewing levels

In each sample, the *XIST* allele-specific expression (*XIST*_ASE_) was used as proxy for XCI skewing levels. *XIST* is uniquely expressed from the Xi, and thus the relative expression of parental alleles within *XIST* transcript is representative of XCI skewing in a bulk sample [7,28,29,70,71]. Within each sample, the *XIST*_ASE_ values (calculated as described above) range from 0 to 1, with 0 or 1 indicating completely skewed XCI (100:0 XCI ratio), and 0.5 indicating balanced inactivation ratio. To be consistent with previous literature [23,28,72], we classified samples with *XIST*_ASE_ ≤0.2 or *XIST*_ASE_ ≥0.8 to have skewed XCI, and samples with 0.2< *XIST*_ASE_ < 0.8 to have random XCI. To have an absolute measure of the magnitude of XCI skewing levels in each sample, the degree of XCI skewing (DS) was calculated from the *XIST*_ASE_ calls. DS is defined as the absolute deviation of *XIST*_ASE_ from 0.5, and it has been similarly been used to assess XCI patterns and XCI status of X-genes [26,73,74]. In each sample, DS was calculated as follows:

$$DS_{s}=|0.5‐XIST_{ASE}|\;Degree\;of\;XCI‐skew\;in\;sample\;s$$ [4]

DS is a proxy for the magnitude of the sample’s XCI-skew. DS values range from 0 to 0.5; 0 indicates random XCI and 0.5 completely skewed XCI. Samples with DS ≥0.3 were classified to have skewed XCI; samples with DS<0.3 to have random XCI patterns. Due to low number of skewed whole-blood samples with available data in other tissues, we excluded our whole-blood estimates from analyses of XCI escape. We found no significant association between *XIST* gene expression levels and DS (Fig A in S1 Appendix).

### Quantification of XCI escape

Bulk samples with random XCI patterns confound mono- and bi-allelic X-linked expression as both X-alleles would be, on overall, expressed. Conversely, in skewed samples (S8 Table) silenced genes will exhibit monoallelic expression while escape genes biallelic expression [8,11,26] (Note A in S1 Appendix). Only genes with RNAseq allelic read depth ≥8 reads were used. Furthermore, to increase the confidence that genotypes were truly heterozygous, we considered only genes whose both haplotypes were detected in the RNA-seq data at least once. We reasoned that variations in DS might influence allelic variation in X-linked aFC leading to biases in escape measurements across samples. To account for this, we implemented a linear model of the sample’s DS as explanatory variable and the genes’ aFCs (as defined above) as response variable using our entire skewed cohort of 166 LCLs, 26 whole-blood, 57 adipose and 64 skin samples. We computed residuals (referred to as ’EscScores’) from the linear model corresponding to the difference between observed (aFC) and predicted response variable. We then rescaled the residuals to be within the [0,1] range via min-max normalization, as follows:

$$EscScore\prime =\frac{EscScore-Min(EscScore)}{Max(EscScore)-Min(EscScore)}Min‐Max\;rescaling$$ [5]

where EscScore′ is the rescaled EscScore (now within the 0–1 range) used for analyses. We verified that as opposed to the raw gene’s aFC values which correlate with the degree of XCI-skew (*ρ* = -0.18; P<2e-16), there was no evidence of correlation between the newly derived EscScore(s) and the degree of XCI-skew in our dataset (*ρ* = 0). This indicates that our procedure of using residuals and normalization generates EscScore(s) robust to variation in XCI-skew across samples removing the dependence between aFC and XCI-skew. To further assess this latter in detail, we grouped our dataset into 3 bins of degree of XCI-skew, and then randomly sampled 200 X-linked genes to check their distribution of average aFC and average EscScore values at the different degrees of XCI-skew. We repeated the random sampling step 3 times (each drawing 200 X-genes) and confirmed that as opposed to aFC, the EscScore is robust to various degree of XCI-skew (Fig B in S1 Appendix).

EscScore 0 and 1 indicate complete monoallelic (silencing) and complete biallelic expression (full escape), respectively. Due to low number of skewed whole-blood samples with available data in other tissues, we excluded our whole-blood estimates from analyses of XCI escape. We detected average EscScore of 0.32, 0.36, and 0.37 across LCLs, adipose and skin samples, respectively, whose median is 0.36. We compared different EscScore cutoffs to the Balaton’s list [30] and found that 0.36 resulted to better reproducibility of existing gene calls (see also Result paragraph 1; Table C in S1 Appendix). We classified genes exhibiting a median (across ≥3 tissue samples) EscScore≥0.36 as escapees in that tissue, while all others as silenced. We show the distribution of median EscScore values of genes with different previously annotated XCI status [30] in Fig 1A. Further, we checked how the EscScore values distributed for genes previously annotated as XCI-silenced: (i) in LCLs, 97% of EscScore values are <0.36; (ii) in adipose, 84% of EscScore values are <0.36; (iii) in skin, 82% of EscScore(s) are <0.36. Altogether these patterns support the suitability of both metric and cutoff used (see also Table C in S1 Appendix).

For analysis of inter-individual variability of escape, genes classified as escapee in at least 1 tissue (median EscScore across skewed tissue samples ≥0.36) and with available EscScore data in ≥10 tissue samples were used. Consistent EscScore in a tissue across individuals was defined when in ≥80% of individuals the gene’s EscScore lay between ±1 standard deviation from the gene’s average EscScore in that tissue (S1 Fig). Otherwise, the gene was deemed variable across individuals (S2 Fig).

### Protein-protein interaction (PPI) network and gene ontology analyses

Genes with EscScore data available in all three tissues and escaping XCI in at least one tissue were analysed for protein-protein interaction (PPI) with a new genome-wide protein interactome database as reference (www.interactome-atlas.org) [32]. PPI network was imported to STRING v.11 [75], and proteins with at least one direct interaction with our genes and a PPI score (edge confidence) ≥0.4 were selected. PPI network was imported to Cytoscape v.3.8.2 [76] for visualization and gene ontology analyses of Biological Processes and REACTOME Pathways using ClueGO v.2.5.8 [77]. A term was considered as significantly enriched at Bonferroni-corrected *P-value* ≤0.01.

## Supporting information

S1 Appendix **Table A.** Summary statistics (mean, median, standard deviation) of EscScore values of genes with different annotated XCI status according to the Balaton’s list. A) LCLs; B) adipose; C) skin. Each statistics is computed across ≥3 tissue samples. **Table B.** Statistical comparison between the EscScore(s) of different gene categories previously annotated. Escapees refer to genes annotated as fully or mostly escaping XCI. For each interrogated gene, the median EscScore value across ≥ 3 tissue samples was used for comparison. Tables report the p-value of the Wilcoxon test between two gene categories A) LCLs; B) adipose; C) Skin. **Table C.** Benchmarking different EscScore cutoffs against the Balaton’s list of XCI status^.^ The EscScore cutoff of 0.36 resulted into both overall higher reproducibility of gene calls and lower discordance with previously annotated XCI status data. **Table D.** Fraction of X-linked genes exhibiting escape (EscScore ≥0.36 in a tissue in a donor) in 1, 2 or all 3 studied tissues in each of the 6 female donors exhibiting skewed XCI in all three studied tissues (LCLs, adipose, skin). **Fig A.** Scatterplot of degree of skewing (y-axis) and XIST gene expression levels (TMM-normalized; x-axis) in LCLs, adipose and skin tissues. There is no evidence of significant association between the two variables in all three tissues (P>0.1). **Fig B.** Assessing the dependence between genes’ aFC and XCI-skew, and between EscScore and XCI-skew in our dataset. Our data were grouped into 3 (nearly same size) bins of average degree of XCI-skew (XCIskew Bin1 < XCIskew Bin2 < XCIskew Bin3). Random sampling of 200 X-genes was then performed three times (A,B,C). At each sampling, the average aFC and average EscScore values per gene (across samples) were calculated within each XCI-skew bin. The EscScore is robust to various degree of XCI-skew (see also Methods). **Fig C.** Relationship between the gene’s tissue-specific EscScore and gene position on chrX (GRCh38). Each dot represents a gene. Red and green dots are escapee and silenced genes, respectively. **Fig D.** Distribution of median EscScore values in each of the three studied tissues (LCLs, adipose, skin). Median values were calculated per gene across ≥3 skewed tissue samples. **Fig E.** Gating strategy for immune cell sorting. Gating strategy used to sort monocytes (CD14+), B (CD14-, CD3-, CD56-, CD20+), NK (CD14-, CD3-, CD20-, CD56+), T-CD4+ (CD14, CD3+, CD8-, CD4+) and T-CD8+ cells (CD14-, CD3+, CD4-, CD8+) from freshly isolated PBMCs from 2 monozygotic twins exhibiting skewed XCI in LCLs.
(PDF)

S1 Table X-linked genes (N = 157) exhibiting escape in at least one of the three studied tissues (LCLs, adipose, skin) in our dataset. The table lists the gene’s EscScore in each tissue, computed as the median EscScore across ≥3 samples. Column 2 indicates whether the escape status of the gene was either known (previously reported) or is a novel call.
(TXT)

S2 Table X-linked genes (N = 213) with EscScore available in all three studied tissues (LCLs, adipose, skin). The table lists the gene’s EscScore in each tissue, computed as the median EscScore across ≥3 samples.
(TXT)

S3 Table X-linked genes (N = 49) escaping XCI in only one of the three studied tissues (LCLs, adipose, skin). This is a subset of S1 Table.
(TXT)

S4 Table Results from ClueGO [77] enrichment analysis of genes in the PPI network. (XLSX)

S5 Table EscScore(s) of (i) *BTK* and *CD99L2* in LCLs, showing consistency across individuals; (ii) *DDX3X*, *KDM6A* and *UBA1*, showing inter-individual variability in LCLs and at least a solid tissue. (TXT)

S6 Table X-linked genes (N = 53) with EscScore available in all five studied immune cell types (Monocytes, B-cells, T-CD4^+^ cells, T-CD8^+^ cells, NK-cells). The table lists the genes’ EscScore in each immune cell type.
(TXT)

S7 Table Genes exhibiting discordant XCI status between the two MZ co-twins. Genes’ EscScore in monocytes, B-cells and T-CD8^+^ cells in each individual are reported.
(XLSX)

S8 Table *XIST* gene expression levels (TMM-normalized), DS, and chrX allelic read depth per tissue sample. The 6 donors examined for intra-individual analysis are annotated.
(XLSX)

S1 Fig Boxplot of gene’s EscScore in each skewed sample (≥10 tissue samples used for this analysis). Plotted are genes classified to have consistent EscScore across individuals. Each green dot is an individual.
(PDF)

S2 Fig Boxplot of gene’s EscScore in each skewed sample (≥10 tissue samples used for this analysis). Plotted are genes classified to have variable EscScore across individuals. Each green dot is an individual.
(PDF)
