# Supplementary material for: Escape from X-inactivation in twins exhibits intra- and inter-individual variability across tissues and is heritable
Source: PLoS Genet. 2023 Feb 21;19(2):e1010556. doi: 10.1371/journal.pgen.1010556 (PMC9942974; doi:10.1371/journal.pgen.1010556)
Supplement: S1 Appendix — Table A. Summary statistics (mean, median, standard deviation) of EscScore values of genes with different annotated XCI status according to the Balaton’s list. A) LCLs; B) adipose; C) skin. Each statistics is computed across ≥3 tissue samples. Table B. Statistical comparison between the EscScore(s) of different gene categories previously annotated. Escapees refer to genes annotated as fully or mostly escaping XCI. For each interrogated gene, the median EscScore value across ≥ 3 tissue samples was used for comparison. Tables report the p-value of the Wilcoxon test between two gene categories A) LCLs; B) adipose; C) Skin. Table C. Benchmarking different EscScore cutoffs against the Balaton’s list of XCI status. The EscScore cutoff of 0.36 resulted into both overall higher reproducibility of gene calls and lower discordance with previously annotated XCI status data. Table D. Fraction of X-linked genes exhibiting escape (EscScore ≥0.36 in a tissue in a donor) in 1, 2 or all 3 studied tissues in each of the 6 female donors exhibiting skewed XCI in all three studied tissues (LCLs, adipose, skin). Fig A. Scatterplot of degree of skewing (y-axis) and XIST gene expression levels (TMM-normalized; x-axis) in LCLs, adipose and skin tissues. There is no evidence of significant association between the two variables in all three tissues (P>0.1). Fig B. Assessing the dependence between genes’ aFC and XCI-skew, and between EscScore and XCI-skew in our dataset. Our data were grouped into 3 (nearly same size) bins of average degree of XCI-skew (XCIskew Bin1 < XCIskew Bin2 < XCIskew Bin3). Random sampling of 200 X-genes was then performed three times (A,B,C). At each sampling, the average aFC and average EscScore values per gene (across samples) were calculated within each XCI-skew bin. The EscScore is robust to various degree of XCI-skew (see also Methods). Fig C. Relationship between the gene’s tissue-specific EscScore and gene position on chrX (GRCh38). Each dot represents a gene. R [file pgen.1010556.s001.pdf]

Antonino Zito[1,#a,#b*], Amy L. Roberts[1¶], Alessia Visconti[1¶], Niccolo' Rossi[1], Rosa Andres-Ejarque[2], Stefano Nardone[3], Julia S. El-Sayed Moustafa[1], Mario Falchi[1], Kerrin S. Small[1*]

[1]Department of Twin Research & Genetic Epidemiology, King's College London, London, UK;

[#]Current Address: [#a]Department of Molecular Biology, Massachusetts General Hospital, Boston, Massachusetts, USA; [#b]Department of Genetics, The Blavatnik Institute, Harvard Medical School, Boston, Massachusetts, USA;

[2]St John's Institute of Dermatology, Faculty of Life Science & Medicine, King's College London, London, UK;

[3]Division of Endocrinology, Diabetes, and Metabolism, Department of Medicine, Beth Israel Deaconess Medical Center, Harvard Medical School, Boston, Massachusetts, USA.

¶ equally contributed

*antonino.zito@kcl.ac.uk (AZ); *kerrin.small@kcl.ac.uk (KSS)

SUPPLEMENTAL DATA

SUPPLEMENTAL NOTES

Note A in S1 Appendix

XIST plays essential roles in XCI [1-3]. *XIST* spreads in *cis* from the Xq, triggering an epigenetic silencing of the X designated for inactivation (Xi) [2,4,5]. *XIST* RNA is exclusively expressed from the Xi [3,6,7], thus the *XIST* allele-specific expression ($XIST_{ASE}$, see Methods) can be used to determine the sample's XCI-skew [8,9]. Genes with Xi-specific expression such as *XIST* are exceptional. As XCI occurs at random within each cell, a somatic tissue with random XCI patterns is a mosaic of cells with either parental X silenced. In such a scenario, all X-genes would exhibit biallelic expression, confounding silenced and escape genes [10]. Conversely, in bulk samples with skewed XCI, most expression will be restricted to one haplotype, enabling distinguishment of monoallelic (XCI-silenced genes) from biallelic (escape genes) expression [7,10,11].

Note B in S1 Appendix

We assessed differences in the incidence of escape between short (Xp) vs long X-arm (Xq). In line with literature [7,12], we found a higher prevalence of escape on Xp (S3 Fig). This phenomenon has biological explanations as (i) Xp-genes have had Y-paralogs; (ii) the centromere might be a 'physical barrier' against the spreading in *cis* of *XIST* from Xq to Xp.

SUPPLEMENTAL TABLES

A

XCI STATUS	MEAN	MEDIAN	SD
SILENCED	0.26	0.21	0.16
VAR.ESCAPEES	0.33	0.29	0.18
ESCAPEES	0.49	0.48	0.21
UNKNOWN STATUS	0.33	0.27	0.2

B

XCI STATUS	MEAN	MEDIAN	SD
SILENCED	0.33	0.27	0.2
VAR.ESCAPEES	0.33	0.3	0.17
ESCAPEES	0.5	0.49	0.21
UNKNOWN STATUS	0.34	0.28	0.21

C

XCI STATUS	MEAN	MEDIAN	SD
SILENCED	0.34	0.29	0.21
VAR.ESCAPEES	0.32	0.28	0.17
ESCAPEES	0.48	0.47	0.21
UNKNOWN STATUS	0.37	0.33	0.21

Table A in S1 Appendix: Summary statistics (mean, median, standard deviation) of EscScore values of genes with different annotated XCI status according to the Balaton's list [13]. A) LCLs; B) adipose; C) skin. Each statistics is computed across ≥3 tissue samples.

A

XCI STATUS	SILENCED	VAR.ESCAPEES	ESCAPEES
SILENCED	*	>0.05	9e-24
VAR.ESCAPEES	>0.05	*	9e-8
ESCAPEES	9e-24	9e-8	*

B

XCI STATUS	SILENCED	VAR.ESCAPEES	ESCAPEES
SILENCED	*	>0.05	4e-14
VAR.ESCAPEES	>0.05	*	1e-4
ESCAPEES	4e-14	1e-4	*

C

XCI STATUS	SILENCED	VAR.ESCAPEES	ESCAPEES
SILENCED	*	>0.05	1e-14
VAR.ESCAPEES	>0.05	*	2e-4
ESCAPEES	1e-14	2e-4	*

Table B in S1 Appendix: Statistical comparison between the EscScore(s) of different gene categories previously annotated [13]. Escapees refer to genes annotated as fully or mostly escaping XCI. For each interrogated gene, the median EscScore value across ≥ 3 tissue samples was used for comparison. Tables report the p-value of the Wilcoxon test between two gene categories A) LCLs; B) adipose; C) Skin.

LCL

EscScore Cutoff	*% escape genes in our data overlapping with Balaton list of escapee genes*	*% silenced genes in our data overlapping with Balaton list of silenced genes*	*% discordant calls (escape here & silenced in Balaton)*	*% genes escaping XCI (tissue prevalence of escape)*
0.31	57%	75%	17%	20%
0.33	59%	75%	12%	18%
0.36	59%	73%	10%	16%

Fat

EscScore Cutoff	*% escape genes in our dataset overlapping with Balaton list of escapee genes*	*% silenced genes in our data overlapping with Balaton list of silenced genes*	*% discordant calls (escape here & silenced in Balaton)*	*% genes escaping XCI (tissue prevalence of escape)*
0.31	28%	78%	47%	41%
0.33	32%	76%	44%	34%
0.36	39%	74%	40%	26%

Skin

EscScore Cutoff	*% escape genes in our data overlapping with Balaton list of escapee genes*	*% silenced genes in our data overlapping with Balaton list of silenced genes*	*% discordant calls (escape here & silenced in Balaton)*	*% genes escaping XCI (tissue prevalence of escape)*
0.31	28%	72%	53%	48%
0.33	33%	73%	45%	37%
0.36	39%	73%	38%	29%

Table C in S1 Appendix: Benchmarking different EscScore cutoffs against the Balaton's list of XCI status [13]. The EscScore cutoff of 0.36 resulted into both overall higher reproducibility of gene calls and lower discordance with previously annotated XCI status data.

DONOR	% ESCAPE IN 1 TISSUE	% ESCAPE IN 2 TISSUES	% ESCAPE IN 3 TISSUES
**DONOR 1**	17.8%	5.6%	1.7%
**DONOR 2**	17.4%	3.5%	3.5%
**DONOR 3**	26.6%	7.8%	7%
**DONOR 4**	34.5%	6%	2%
**DONOR 5**	27.5%	3.8%	7.6%
**DONOR 6**	30%	2.6%	2.6%

Table D in S1 Appendix: Fraction of X-linked genes exhibiting escape (EscScore $\geq$0.36 in a tissue in a donor) in 1, 2 or all 3 studied tissues in each of the 6 female donors exhibiting skewed XCI in all three studied tissues (LCLs, adipose, skin).

Tables S1, S2, S3, S4, S5, S6,S7,S8 are described below and provided as separate individual files:

S1 Table: X-linked genes (N=157) exhibiting escape in at least one of the three studied tissues (LCLs, adipose, skin) in our dataset. The table lists the gene's EscScore in each tissue, computed as the median EscScore across ≥3 samples. Column 2 indicates whether the escape status of the gene was either known (previously reported) or is a novel call.

S2 Table: X-linked genes (N=213) with EscScore available in all three studied tissues (LCLs, adipose, skin). The table lists the gene's EscScore in each tissue, computed as the median EscScore across ≥3 samples.

S3 Table: X-linked genes (N=49) escaping XCI in only one of the three studied tissues (LCLs, adipose, skin). This is a subset of S1 Table.

S4 Table: Results from ClueGO [14] enrichment analysis of genes in the PPI network

S5 Table: EscScore(s) of (i) *BTK* and *CD99L2* in LCLs, showing consistency across individuals; (ii) *DDX3X*, *KDM6A* and *UBA1*, showing inter-individual variability in LCLs and at least a solid tissue.

S6 Table: X-linked genes (N=53) with EscScore available in all five studied immune cell types (Monocytes, B-cells, T-CD4$^+$ cells, T-CD8$^+$ cells, NK-cells). The table lists the genes' EscScore in each immune cell type.

S7 Table. Genes exhibiting discordant XCI status between the two MZ co-twins. Genes' EscScore in monocytes, B-cells and T-CD8$^+$ cells in each individual are reported.

S8 Table: *XIST* gene expression levels (TMM-normalized), DS, and chrX allelic read depth per tissue sample. The 6 donors examined for intra-individual analysis are annotated.

SUPPLEMENTAL FIGURES

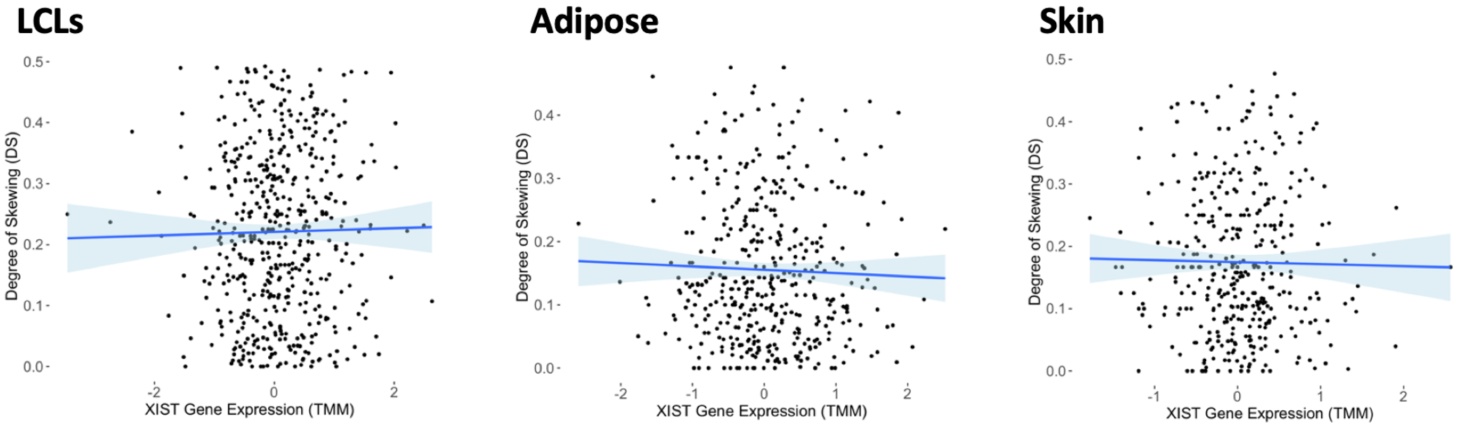

Fig A in S1 Appendix: Scatterplot of degree of skewing (y-axis) and XIST gene expression levels (TMM-normalized; x-axis) in LCLs, adipose and skin tissues. There is no evidence of significant association between the two variables in all three tissues (P>0.1).

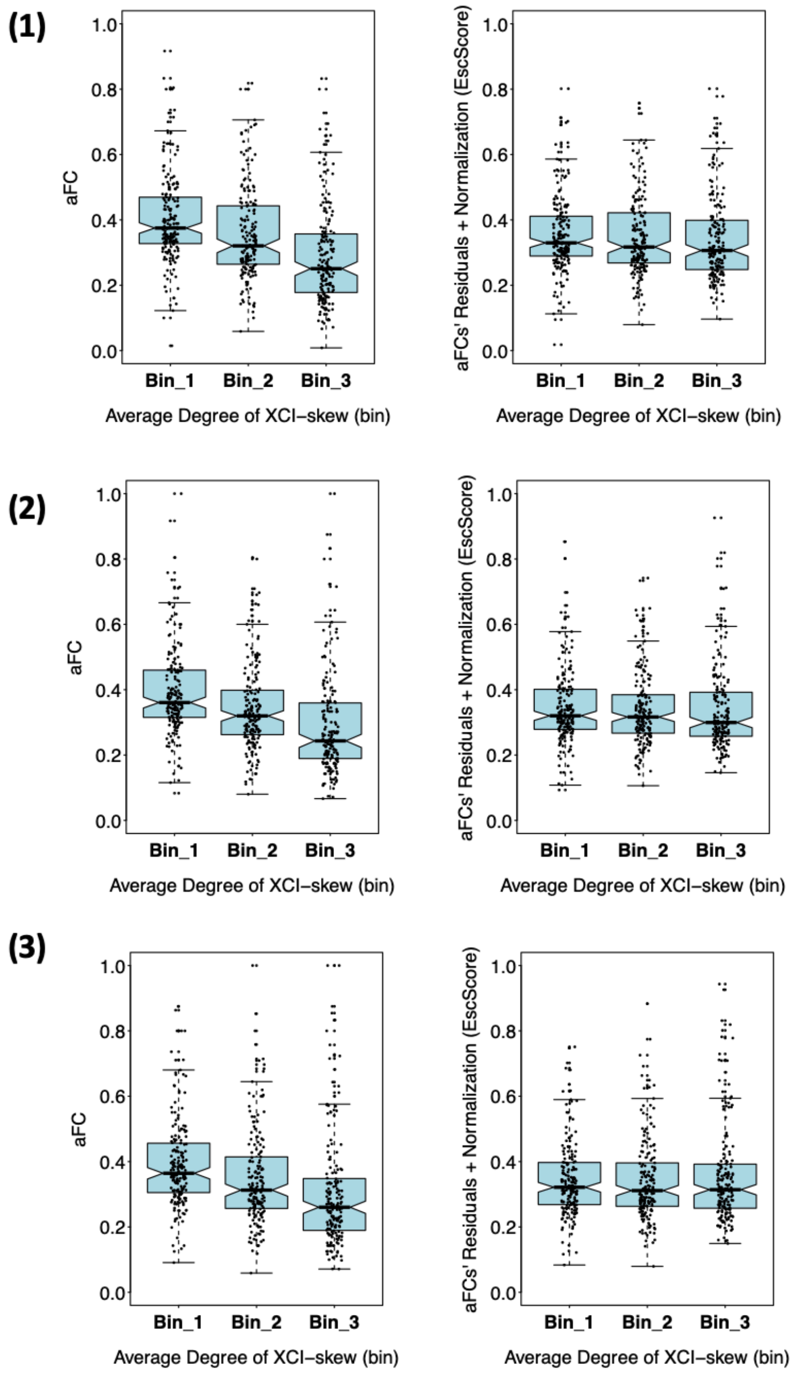

Fig B in S1 Appendix: Assessing the dependence between genes' aFC and XCI-skew, and between EscScore and XCI-skew in our dataset. Our data were grouped into 3 (nearly same size) bins of degree of XCI-skew (XCIskew Bin1 < XCIskew Bin2 < XCIskew Bin3). Random sampling of 200 X-genes was then performed three times (1,2,3). At each sampling, the average aFC and average EscScore values per gene (across samples) were calculated within each XCI-skew bin. The EscScore is robust to various degree of XCI-skew (see also Methods).

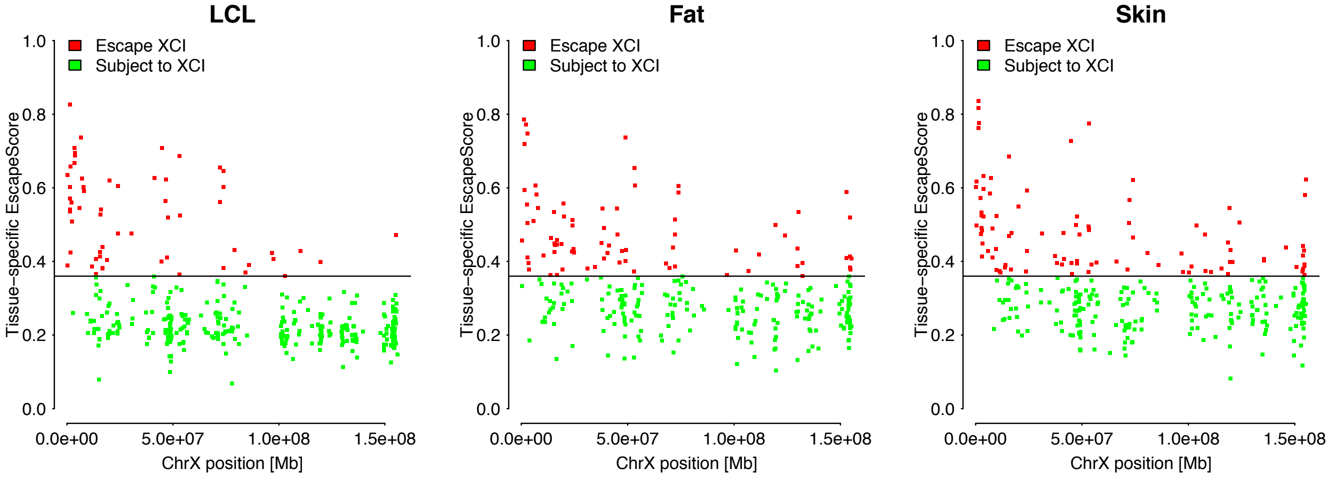

Fig C in S1 Appendix: Relationship between the gene's tissue-specific EscScore and gene position on chrX (GRCh38). Each dot represents a gene. Red and green dots are escapee and silenced genes, respectively.

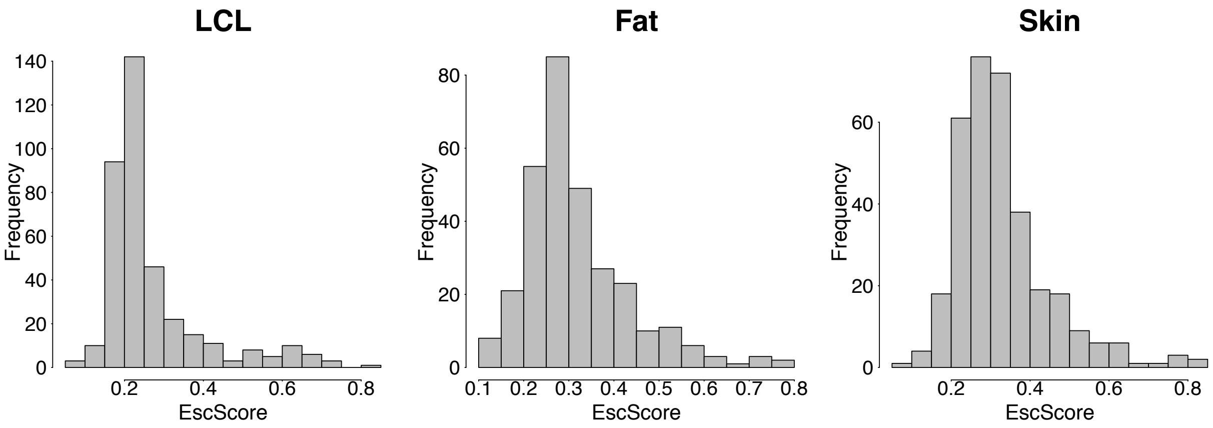

Fig D in S1 Appendix: Distribution of median EscScore values in each of the three studied tissues (LCLs, adipose, skin). Median values were calculated per gene across ≥3 skewed tissue samples.

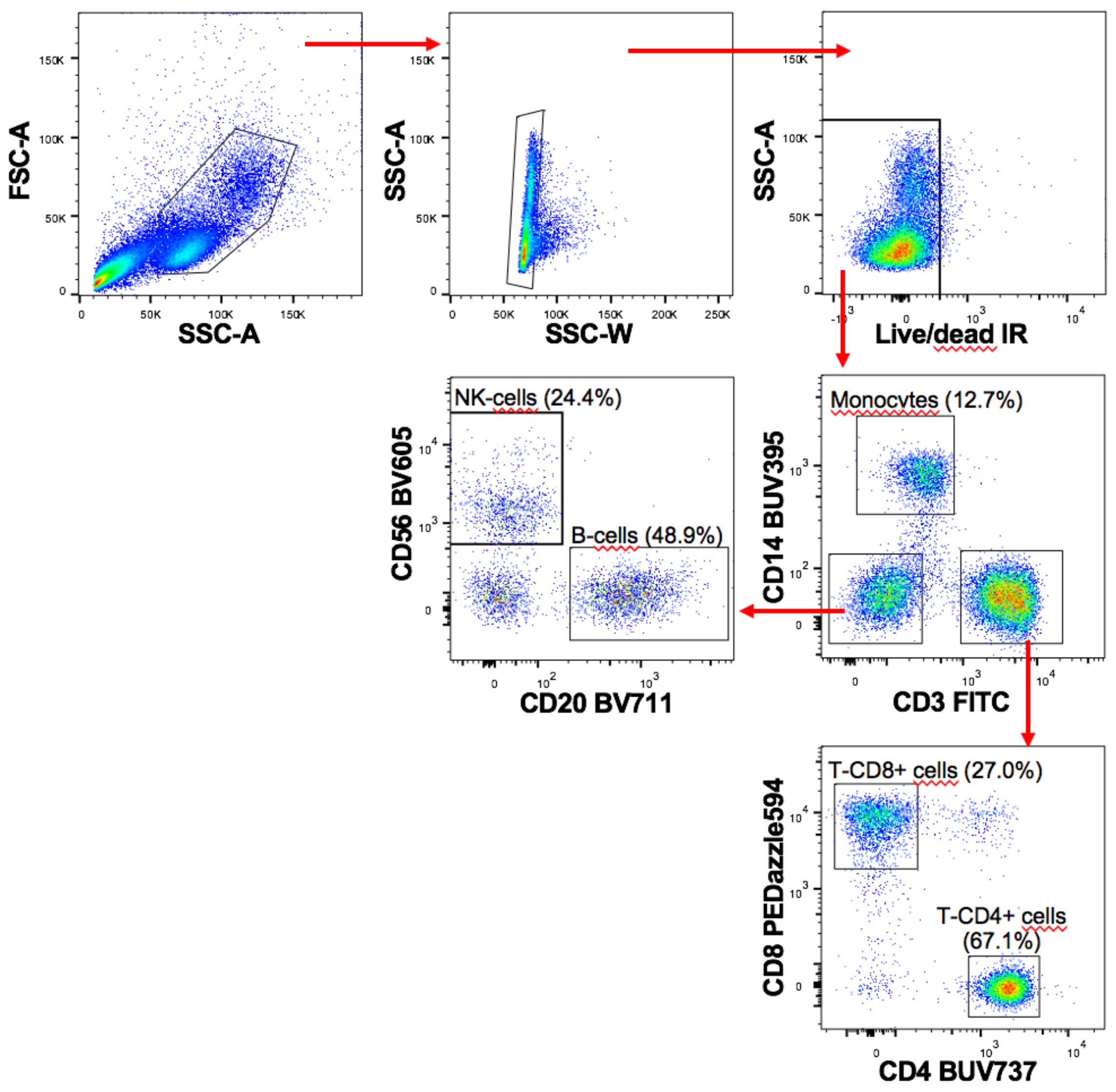

Fig E in S1 Appendix: Gating strategy for immune cell sorting. Gating strategy used to sort monocytes (CD14+), B (CD14-, CD3-, CD56-, CD20+), NK (CD14-, CD3-, CD20-, CD56+), T-CD4+ (CD14, CD3+, CD8-, CD4+) and T-CD8+ cells (CD14-, CD3+, CD4-, CD8+) from freshly isolated PBMCs from 2 monozygotic twins exhibiting skewed XCI in LCLs.

S1 Fig.pdf and S2 Fig.pdf are described below and provided as separate files:

S1 Fig: Boxplot of gene's EscScore in each skewed sample (≥10 tissue samples used for this analysis). Plotted are genes classified to have consistent EscScore across individuals. Each green dot is an individual.

S2 Fig: Boxplot of gene's EscScore in each skewed sample (≥10 tissue samples used for this analysis). Plotted are genes classified to have variable EscScore across individuals. Each green dot is an individual.

Reference

1. Penny GD, Kay GF, Sheardown SA, Rastan S, Brockdorff N. Requirement for Xist in X chromosome inactivation. Nature. 1996;379(6561):131-7.

2. Simon MD, Pinter SF, Fang R, Sarma K, Rutenberg-Schoenberg M, Bowman SK, et al. High-resolution Xist binding maps reveal two-step spreading during X-chromosome inactivation. Nature. 2013;504(7480):465-9.

3. Brown CJ, Ballabio A, Rupert JL, Lafreniere RG, Grompe M, Tonlorenzi R, et al. A gene from the region of the human X inactivation centre is expressed exclusively from the inactive X chromosome. Nature. 1991;349(6304):38-44.

4. Engreitz JM, Pandya-Jones A, McDonel P, Shishkin A, Sirokman K, Surka C, et al. The Xist lncRNA exploits three-dimensional genome architecture to spread across the X chromosome. Science. 2013;341(6147):1237973.

5. Pinter SF, Sadreyev RI, Yildirim E, Jeon Y, Ohsumi TK, Borowsky M, et al. Spreading of X chromosome inactivation via a hierarchy of defined Polycomb stations. Genome Res. 2012;22(10):1864-76.

6. Brown CJ, Hendrich BD, Rupert JL, Lafreniere RG, Xing Y, Lawrence J, et al. The human XIST gene: analysis of a 17 kb inactive X-specific RNA that contains conserved repeats and is highly localized within the nucleus. Cell. 1992;71(3):527-42.

7. Tukiainen T, Villani AC, Yen A, Rivas MA, Marshall JL, Satija R, et al. Landscape of X chromosome inactivation across human tissues. Nature. 2017;550(7675):244-8.

8. Rupert JL, Brown CJ, Willard HF. Direct detection of non-random X chromosome inactivation by use of a transcribed polymorphism in the XIST gene. Eur J Hum Genet. 1995;3(6):333-43.

9. Zito A, Davies MN, Tsai PC, Roberts S, Andres-Ejarque R, Nardone S, et al. Heritability of skewed X-inactivation in female twins is tissue-specific and associated with age. Nat Commun. 2019;10(1):5339.

10. Cotton AM, Ge B, Light N, Adoue V, Pastinen T, Brown CJ. Analysis of expressed SNPs identifies variable extents of expression from the human inactive X chromosome. Genome Biol. 2013;14(11):R122.

11. Carrel L, Willard HF. X-inactivation profile reveals extensive variability in X-linked gene expression in females. Nature. 2005;434(7031):400-4.

12. Carrel L, Brown CJ. When the Lyon(ized chromosome) roars: ongoing expression from an inactive X chromosome. Philos Trans R Soc Lond B Biol Sci. 2017;372(1733).

13. Balaton BP, Cotton AM, Brown CJ. Derivation of consensus inactivation status for X-linked genes from genome-wide studies. Biol Sex Differ. 2015;6:35.

14. Bindea G, Mlecnik B, Hackl H, Charoentong P, Tosolini M, Kirilovsky A, et al. ClueGO: a Cytoscape plug-in to decipher functionally grouped gene ontology and pathway annotation networks. Bioinformatics. 2009;25(8):1091-3.
