# Supplementary material for: Escape from X-inactivation in twins exhibits intra- and inter-individual variability across tissues and is heritable
Source: PLoS Genet. 2023 Feb 21;19(2):e1010556. doi: 10.1371/journal.pgen.1010556 (PMC9942974; doi:10.1371/journal.pgen.1010556)
Supplement: S1 Fig — Plotted are genes classified to have consistent EscScore across individuals. Each green dot is an individual. (PDF) [file pgen.1010556.s010.pdf]

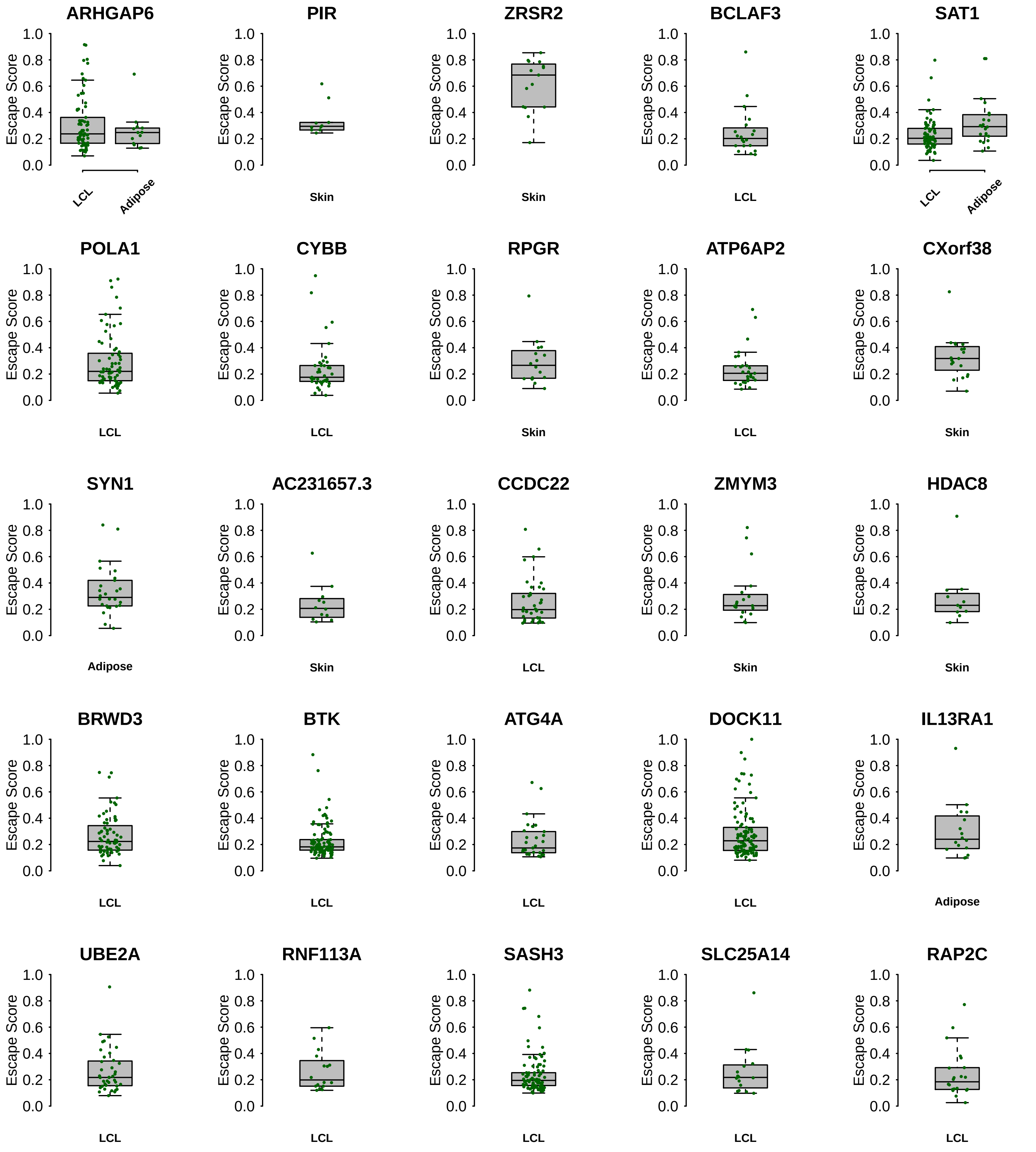
ARHGAP6
PIR
ZRSR2
BCLAF3
SAT1
POLA1
CYBB
RPGR
ATP6AP2
CXorf38
SYN1
AC231657.3
CCDC22
ZMYM3
HDAC8
BRWD3
BTK
ATG4A
DOCK11
IL13RA1
UBE2A
RNF113A
SASH3
SLC25A14
RAP2C
Escape Score
0.0
0.2
0.4
0.6
0.8
1.0
LCL
Adipose
Skin

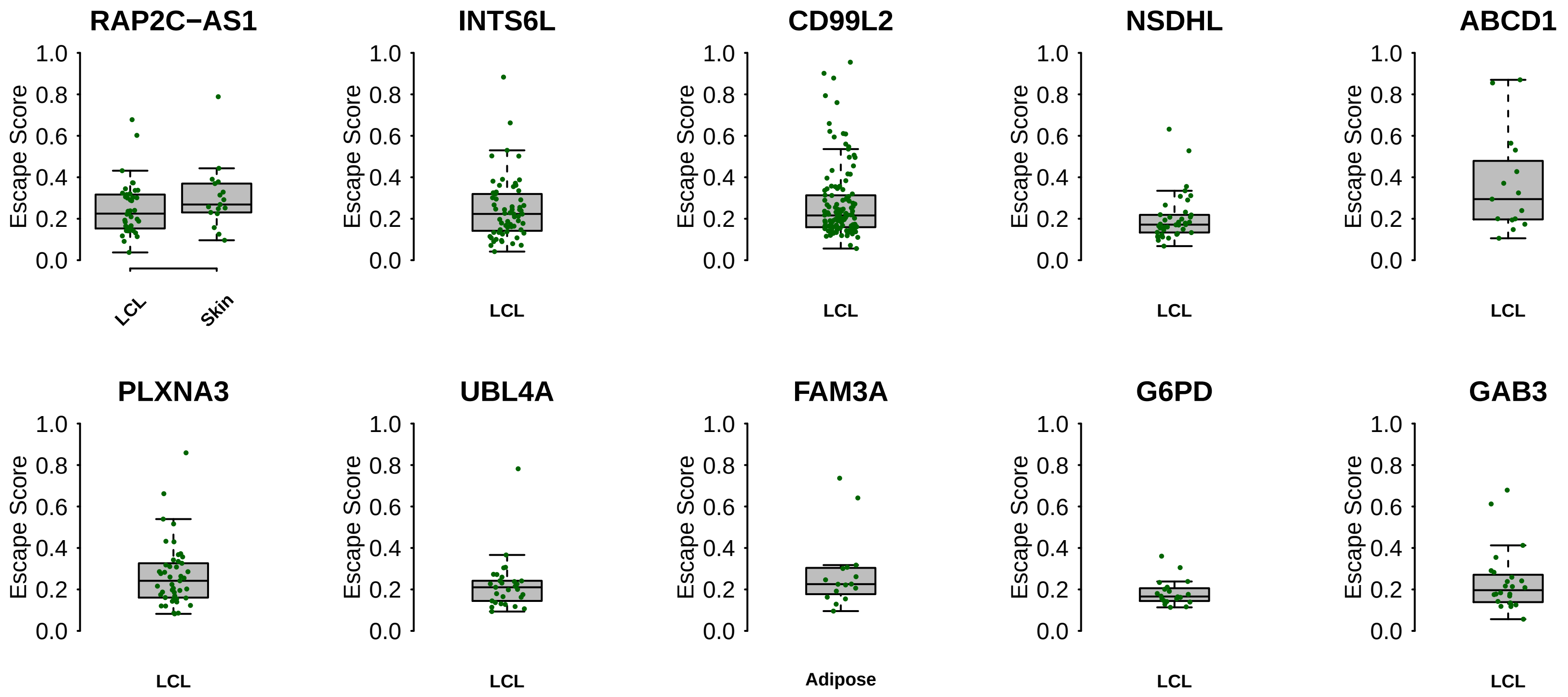

RAP2C−AS1
INTS6L
CD99L2
NSDHL
ABCD1
PLXNA3
UBL4A
FAM3A
G6PD
GAB3
Escape Score
0.0
0.2
0.4
0.6
0.8
1.0
LCL
Skin
Adipose
