# Supplementary figures and images for: Escape from X-inactivation in twins exhibits intra- and inter-individual variability across tissues and is heritable
Source: PLoS Genet. 2023 Feb 21;19(2):e1010556. doi: 10.1371/journal.pgen.1010556 (PMC9942974; doi:10.1371/journal.pgen.1010556)

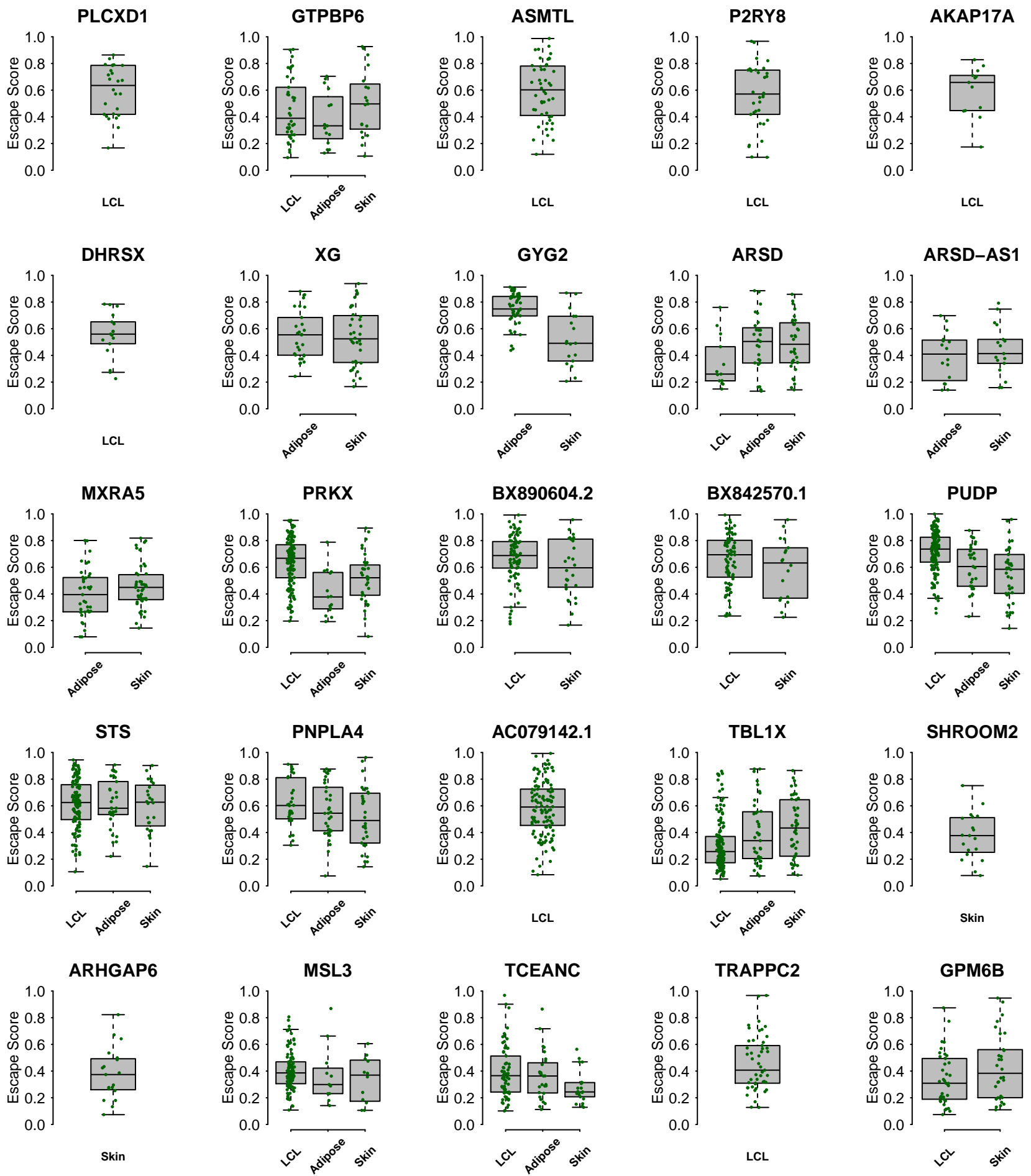

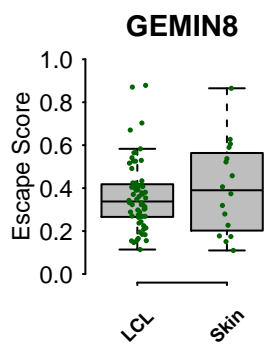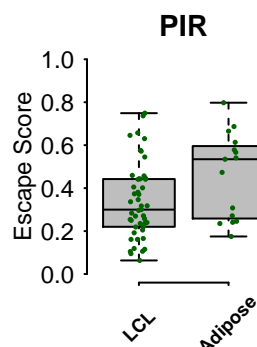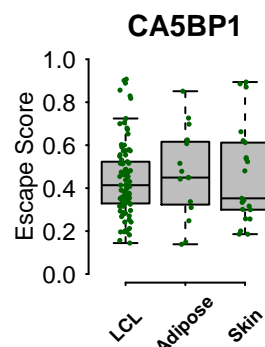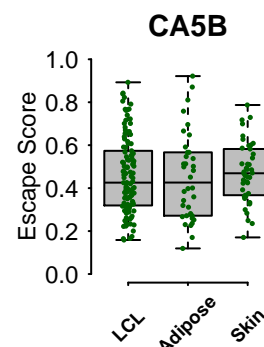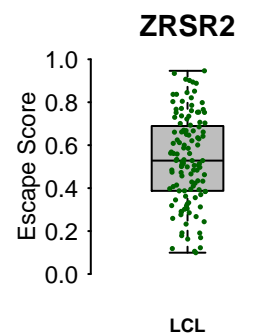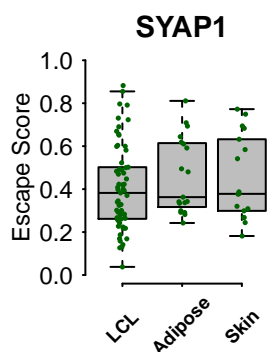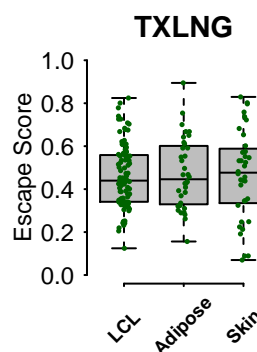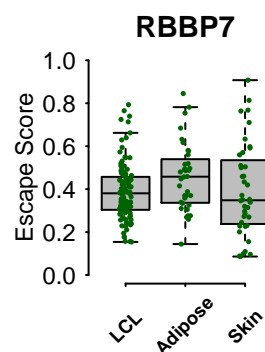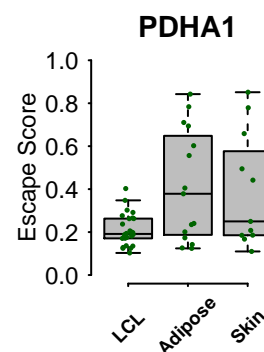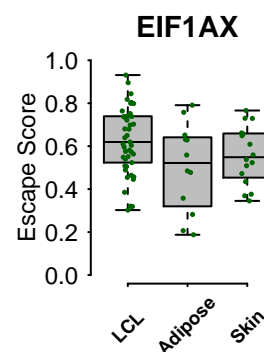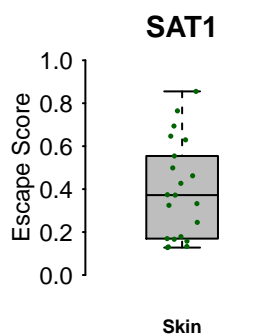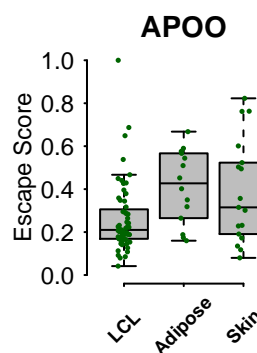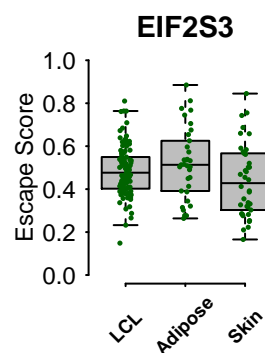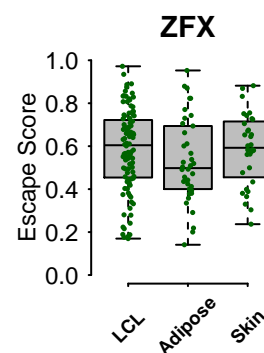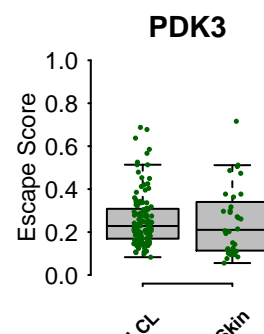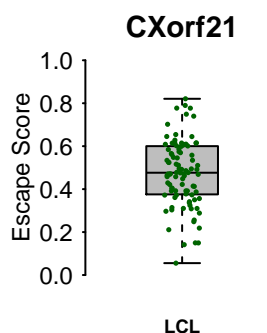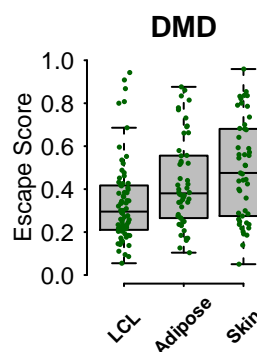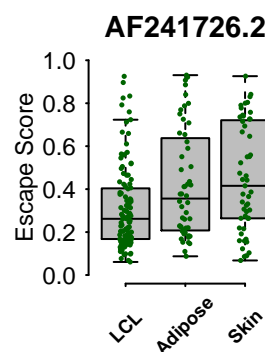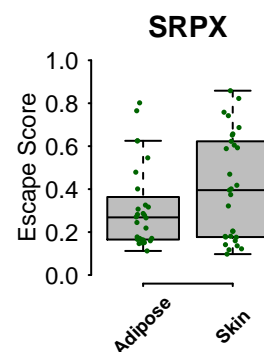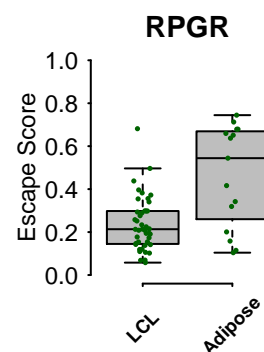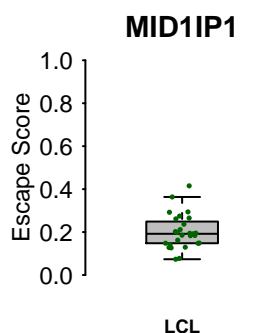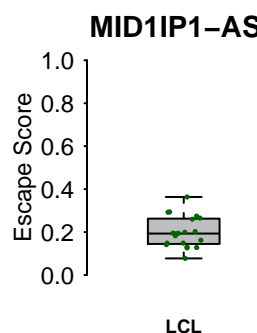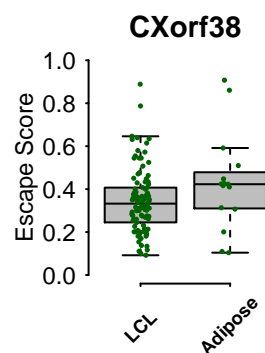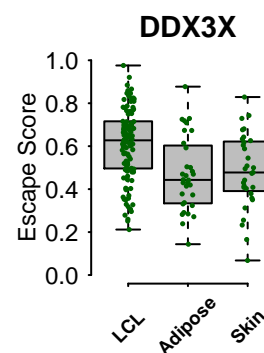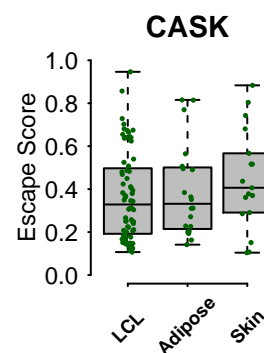

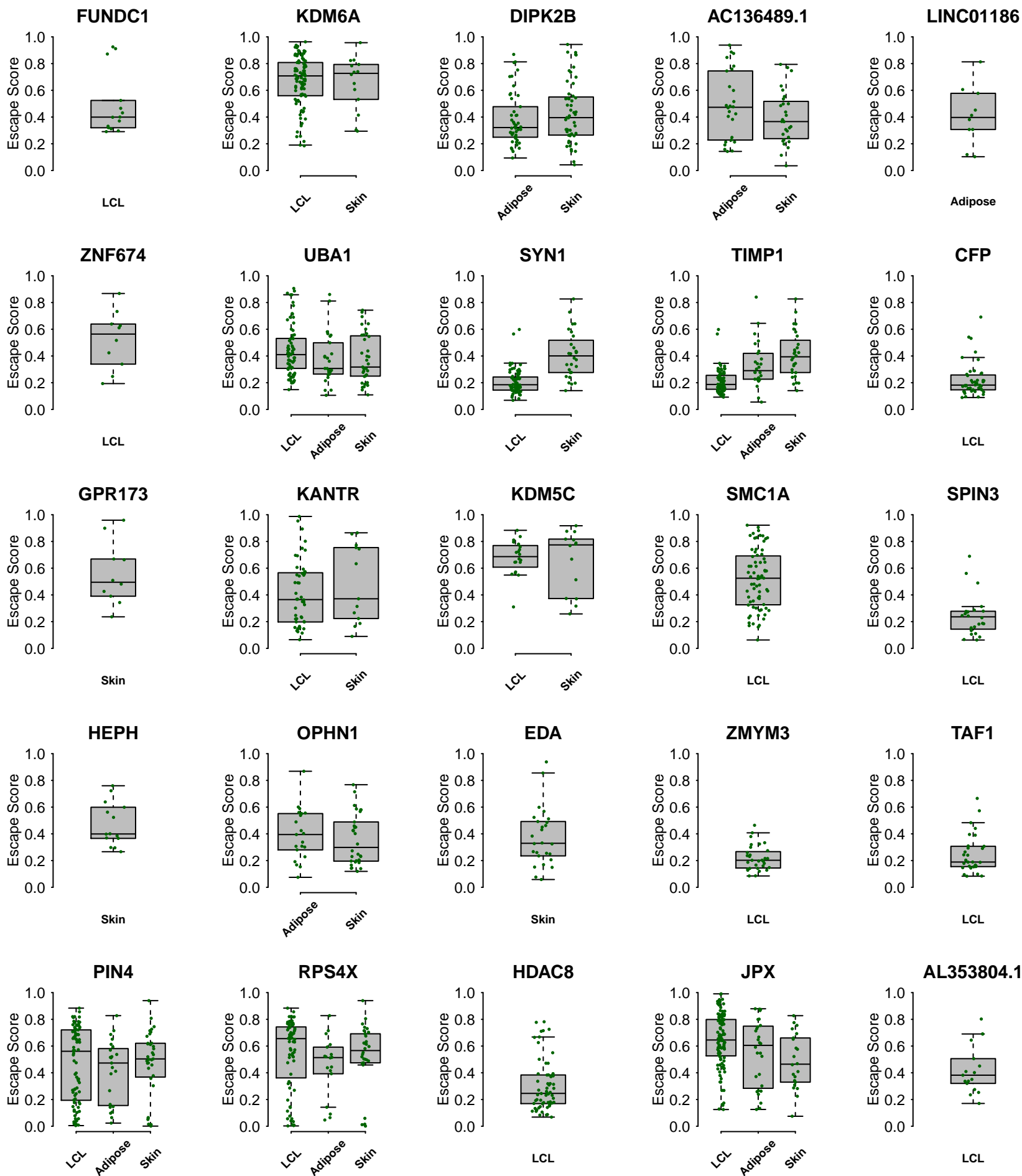

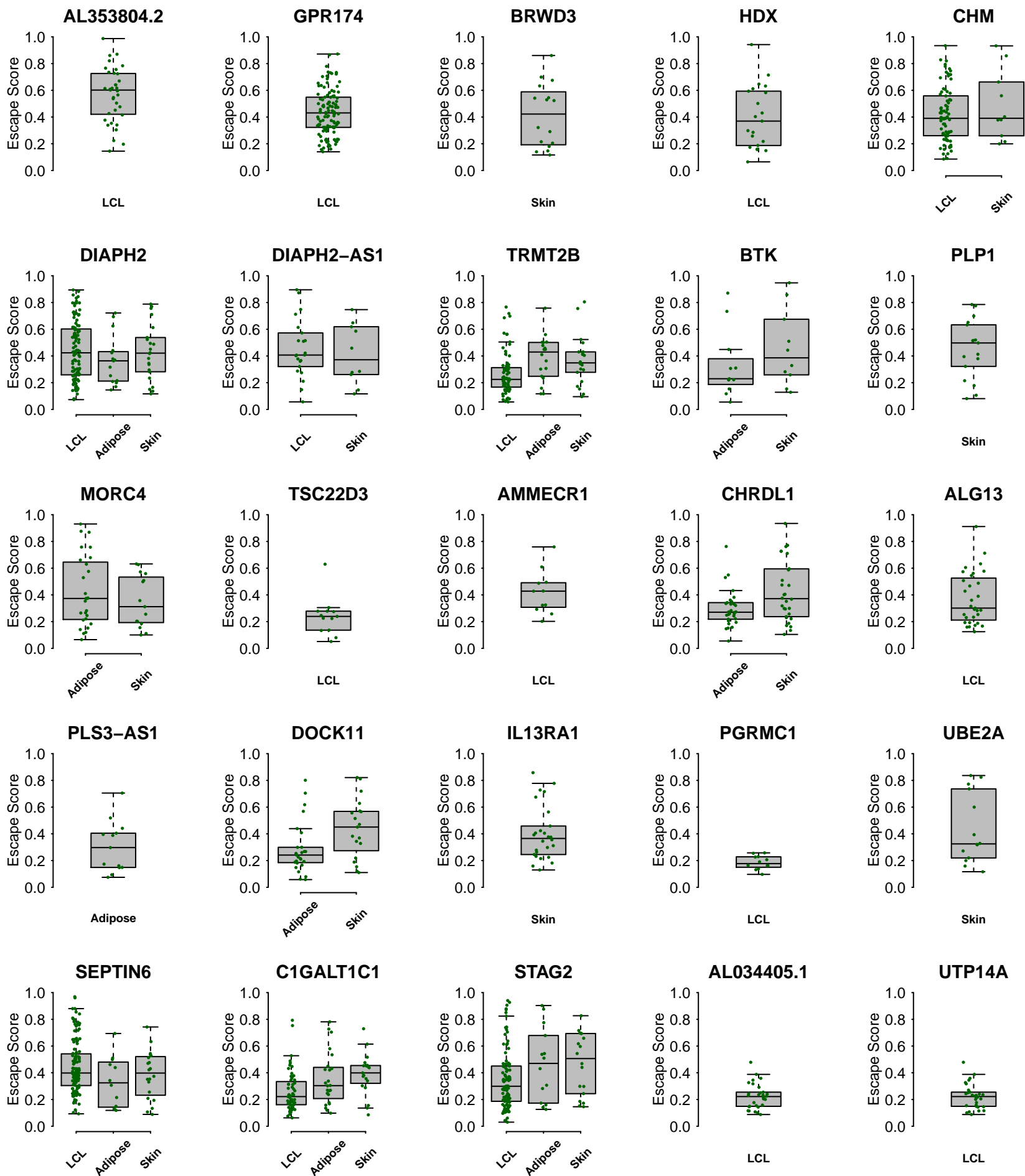

**RAP2C-AS1**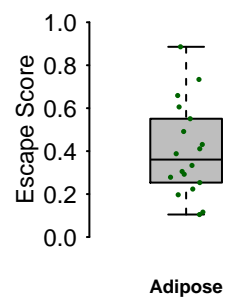**INTS6L**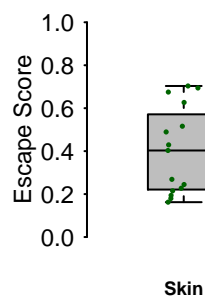**CD99L2**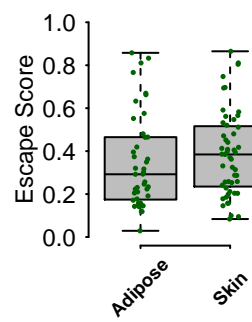**NSDHL**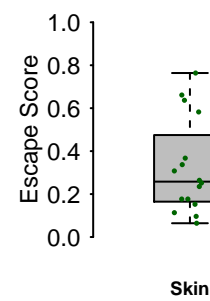**ABCD1**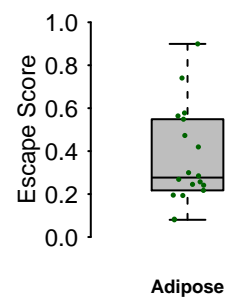**L1CAM**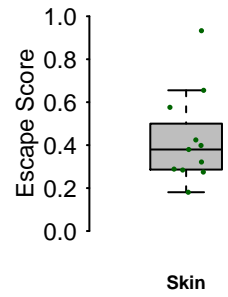**DNASE1L1**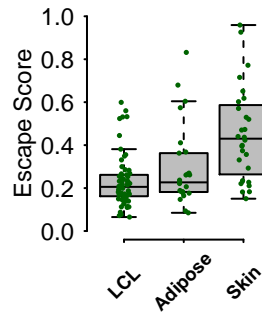**PLXNA3**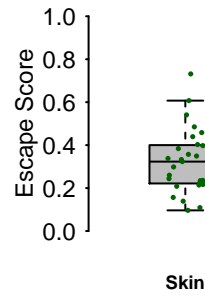**UBL4A**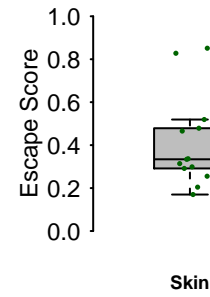**FAM3A**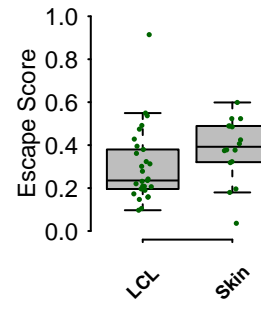**GAB3**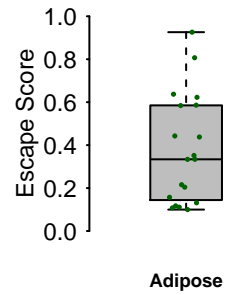**MPP1**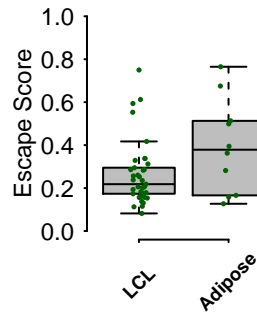**CLIC2**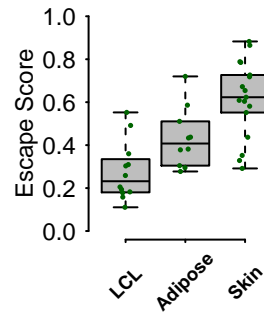

Supplement: S2 Fig — Plotted are genes classified to have variable EscScore across individuals. Each green dot is an individual. (PDF) [file pgen.1010556.s011.pdf]
